# Lithic usewear confirms the function of Wilamaya Patjxa projectile points

**DOI:** 10.1038/s41598-023-45743-7

**Published:** 2023-11-03

**Authors:** Ashley Smallwood, Randall Haas, Thomas Jennings

**Affiliations:** 1 University of Louisville, Louisville, USA; 2 University of Wyoming, Laramie, USA

**Keywords:** Behavioural ecology, Psychology and behaviour

## Abstract

Approximately 9000 years ago at the Andean highland site of Wilamaya Patjxa, forager communities interred female and male individuals with projectile points, suggesting that large-mammal hunting may have been a gender neutral activity among that community. We report a lithic usewear analysis, which confirms that the ostensible projectile points were indeed used as projectile points. The data further reveal evidence of cutting and hide scraping consistent with animal processing activities. A new radiocarbon date shows that the female and male individuals were contemporaries, or nearly so, sometime between 9.0 and 8.7 cal. ka. These findings support a model of early subsistence practices in which both female and male individuals at Wilamaya Patjxa hunted large mammals.

## Introduction

**Figure 1:**
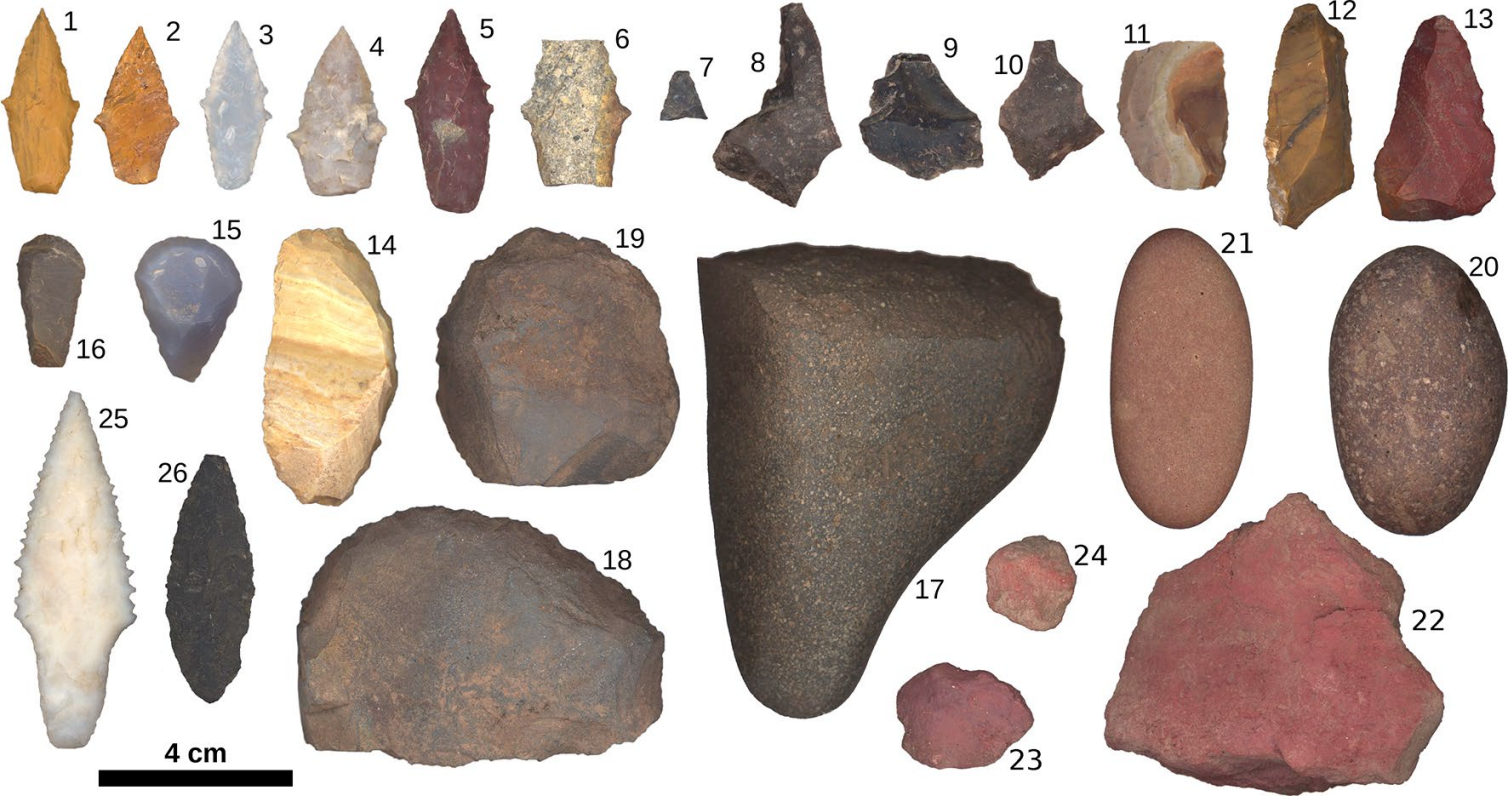
Flaked stone tools associated with Wilamaya Patjxa Individuals 1 and 6. Artifacts 1–24 are associated with WMP 6, the female individual, artifacts 25 and 26 are associated with the WMP1, the male individual. Artifacts 1–7, 25, and 26 are morphologically identified as projectile points. Artifacts 1–14 and 25–26 are the subject of usewear analysis reported here to evaluate tool function.

The extent to which subsistence labor was gendered among early forager societies remains unclear^1–5^. Forager ethnography suggests that large-mammal hunting was the purview of males^6^, but archaeologists have long cautioned against projection of recent forager behavior onto the past^7^. Empirical and theoretical observations identify several socio-ecological conditions that would tend to favor hunting by female individuals including small-animal hunting as a risk-mitigation strategy^8^, communal hunting of large mammals^9^, geographic proximity of large mammals to camp^10^, the use of atlatl technology^3^, and heavy economic dependence on large mammals^11^. The in situ discovery of seven projectile points interred with an adult female 9000 years ago in the Andean highland site of Wilamaya Patjxa (Fig. 1) favors a model of female hunters in that socio-ecological context^12^.

Hunting activities at Wilamaya Patjxa centered on large mammals, as indicated by several lines of evidence. First, the site’s faunal assemblage contains abundant, burnt large-mammal bone, including camelid, deer, and indeterminate large-mammal bone^12^. Small-mammal, bird, and fish remains are notably absent. Second, Andean rock art imagery commonly shows individuals hunting large mammals—camelids and deer—with atlatl technology^13–15^. Third, and more generally, an extensive meta-analysis of projectile technology among ethnographic groups throughout the Americas shows that large-mammal hunting is the major driver of stone-projectile use^16^. Finally, stone projectiles are commonly found in archaeological association with large mammal remains and hunting features throughout the Americas^17–19^. Although lithic projectile points were commonly used for inter-personal violence as well^20–22^, this does not preclude their use in hunting. The sum of evidence indicates that large mammals were the major target of these Andean hunters, suggesting that the Wilamaya Patjxa female was a large-mammal hunter.

Although the female-hunter interpretation offers a parsimonious explanation for the archaeological observations, alternative explanations remain plausible, demanding further investigation. A second viable explanation for the co-occurrence of projectile points with female individuals is that the artifacts were not projectile points at all but rather knives used in domestic contexts^23^. A third possibility is that the objects were symbolic offerings from other community members, presumably male, to honor the deceased female or provide her with hunting tools for the afterlife^2^.

Lithic usewear analysis presents a method for evaluating the competing projectile, knife, and offering hypotheses. The different mechanisms of use entail distinct usewear patterns, or lack thereof, which we evaluate for the projectile points associated with the female and male burials at Wilamaya Patjxa. In addition, we examine usewear on other tool forms to gain insight into the functionality of the entire toolkit associated with the female individual. Last, we assess the contemporaneity of the two individuals by presenting a new radiocarbon determination on bone collagen.

## Results

**Figure 2:**
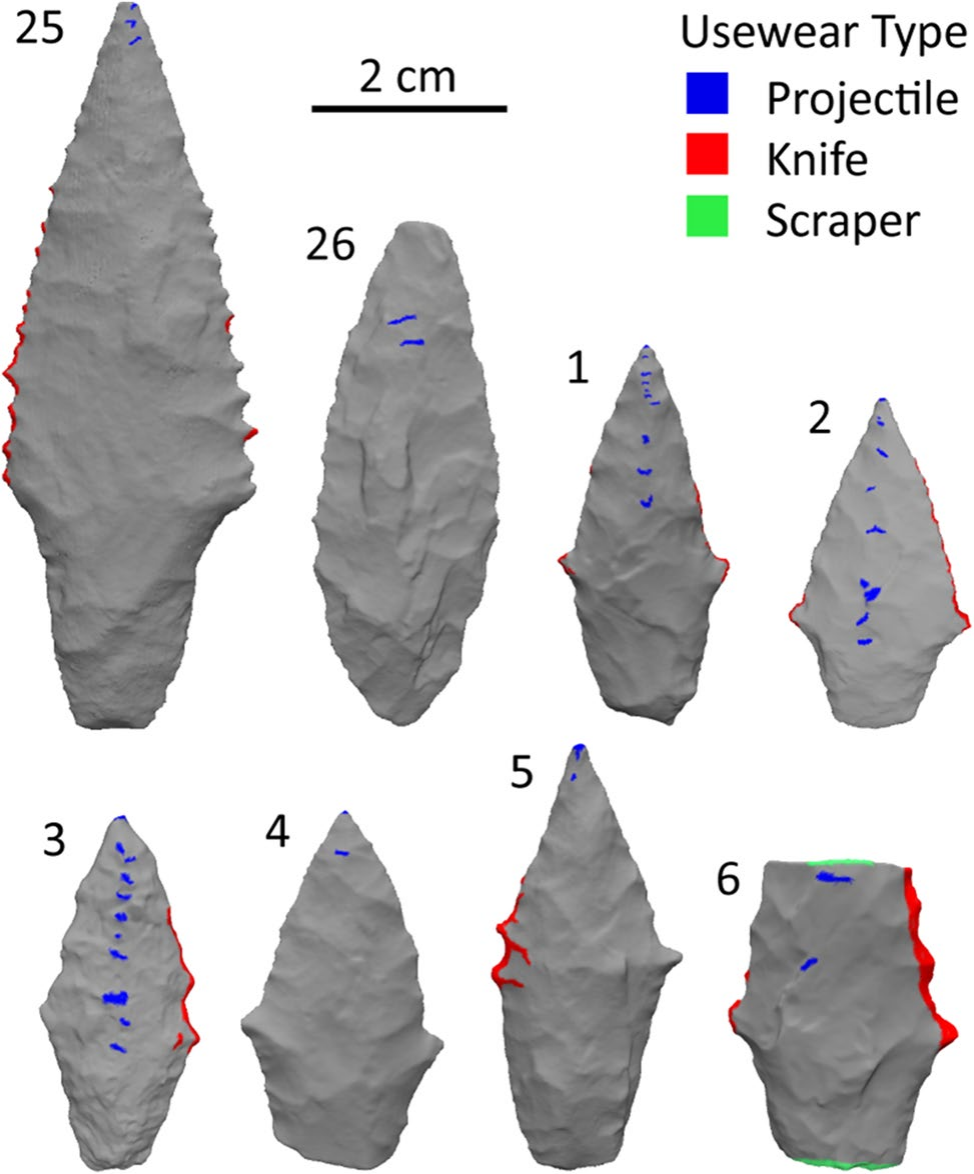
Visual summary of usewear results. All artifacts morphologically identified as projectile points show evidence of projectile wear (blue). All but two—artifacts 4 and 26—show evidence of knife wear (red). Additional data visualizations presented in Supplementary Information: Usewear Data.

**Figure 3:**
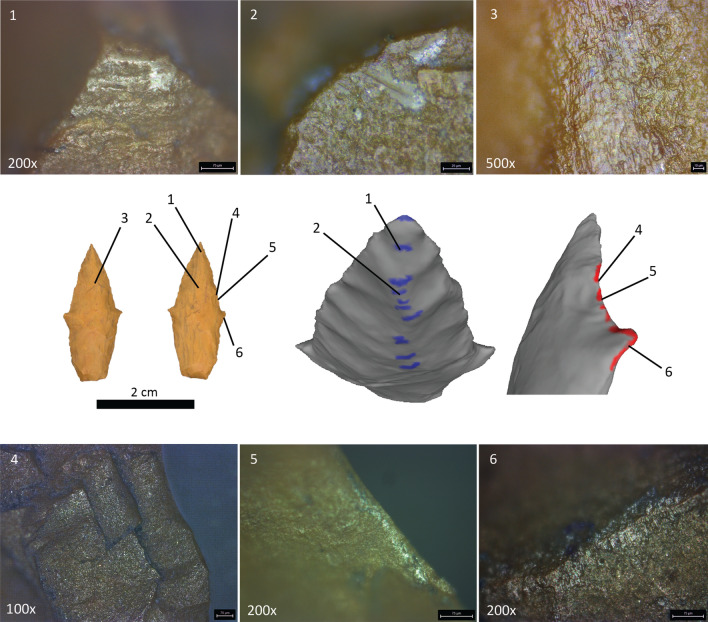
Example of artifact usewear. Artifact 1, a projectile point associated with WMP 6. (1) Polish observed on high, distal aspects of the arrises, indicating projectile use. (2, 3) Striations oriented parallel to long-axis. (4) Microchipping, (5) polish, and (6) striations observed on the distal and proximal aspects of flake scars along the blade margins and ears, indicating knife use. Data plates for other artifacts are presented in Supplementary Information: Usewear Data.

Microscopic examination was performed on 18 artifacts, including all nine projectile points. Both projectile points associated with WMP 1, the male individual, exhibit usewear on flake-scar ridges and distal-facing facets concentrated along the center axis of both blade faces (Figs. 2 and 3; Table 1). Artifact 25 also exhibits usewear along the blade margins including both proximal and distal aspects. These observations indicate that both artifacts associated with the male individual were used as projectile points, and one doubled as a knife.

**Table 1:** Artifacts associated with the Wilamaya Patjxa burials^12^ and usewear observations.

ID	Fig. ID	Context	Morphology	Material	Length (mm)	Width (mm)	Thick. (mm)	Mass (g)	Usewear
913	25	WMP 1	Point	Chert	73.7	25.7	7.8	12.45	Cutting, projectile
912	26		Point	Volcanic	51.1	18.4	6.1	5.04	Projectile
944	1	WMP 6	Point	Chert	38.3	17.0	6.1	3.64	Cutting, projectile
945	2		Point	Chert	32.4	17.5	3.5	1.52	Cutting, projectile
946	3		Point	Chert	34.7	14.7	4.8	2.34	Cutting, projectile
948	4		Point	Chert	35.3	20.4	5.7	3.39	Projectile
947	5		Point	Chert	42.1	18.3	7.0	4.88	Cutting, projectile
949	6		Point mid-section	Chert	29.7	22.9	7.9	4.26	Cutting, projectile, scraping
950	7		Point tip	Chert	10.4	10.6	3.8	0.31	Projectile
941b	8		Flake	Chert	42.0	27.1	7.3	5.15	Scraping
941c	9		Flake	Chert	26.0	26.1	3.6	2.10	Indeterminate
941d	10		Flake	Chert	43.0	25.4	7.3	7.40	None
941a	11		Modified flake	Chert	31.9	23.6	7.0	4.91	Scraping
939	12		Modified flake	Chert	46.7	21.7	6.4	5.30	Cutting
940	13		Modified flake	Chert	42.2	24.7	7.3	7.40	Scraping
938	14		Backed knife	Chert	57.8	28.9	9.0	14.20	Cutting
937	15		Scraper	Chalcedony	32.0	22.9	10.4	7.81	Scraping
936	16		Scraper	Chert	27.5	15.3	6.5	2.44	Scraping
952	17		Scraper	Volcanic	111.5	80.1	36.6	342.00	Not examined
943	18		Scraper	Volcanic	82.0	53.6	27.6	184.14	Not examined
942	19		Scraper	Volcanic	53.9	58.3	25.1	128.09	Not examined
953	20		Hammer	Volcanic	59.6	39.0	26.2	85.41	Not examined
954	21		Cobble	Volcanic	61.8	30.1	12.9	37.70	Not examined
951	22		Ocher	Hematite	72.2	62.3	22.2	118.30	NA
951	23		Ocher	Hematite	28.9	22.1	9.5	7.80	NA
951	24		Ocher	Hematite	19.9	19.9	10.8	3.58	NA

Of the seven artifacts morphologically identified as projectile points in the WMP 6 assemblage, all seven exhibit polish on the tip and distal-facing facets of the blade center axis with striae oriented parallel to the long-axis (see Fig. 2). These traces are concentrated on the blades and are minimal or absent on the stem faces, terminating just below lateral ears as a result of shielding by the hafting mechanism, suggesting that wear is not incidental to transport (cf.,^24^). All but two points exhibit polish and striations along blade margins including both the distal and proximal aspects of point ears. Artifact 6—a projectile point mid-section—also acquired extensive polish and striae along the snapped margins, suggesting scraping action against a hard material such as bone^25,26^. These observations indicate that the points were all used as projectiles and often as knives. The usewear on the snapped point indicates that points were occasionally repurposed as scrapers.

Seven of the nine unifacial artifacts examined show signs of use. One flake and two modified flakes were used for expedient scraping tasks. The flake’s concave edge acquired isolated polish oriented perpendicular to the working edge in both low and high areas. The modified flakes were both used to scrape hard material, but one acquired bimarginal stepped microchipping with polish in negative flake scars and on high arrises of the flake margin indicating contact with bone and soft material, likely meat^26^. One modified flake and a backed knife were used for cutting, contacting bone and meat. The modified flake has two flake margins, one with scalar microchipping and polish where high arrises intersect the cutting edge and the opposing convex margin with polish directly on the otherwise unmodified edge^27,28^. The artifact identified morphologically as a backed knife exhibits polish, scalar flaking, and striae oblique to the retouched concave lateral flake margin suggesting use in butchering^26,29^. Bit edges of the two endscrapers acquired intensive polish, rounding, pitting, and striae perpendicular to the scraping edge—characteristics experimentally associated with the processing of dry hides^30–32^.

A previous attempt at radiocarbon dating the two individuals produced two collagen dates from WMP 6 but failed to produce a date on WMP 1. A second attempt on WMP 1 bone collagen was successful, producing an age estimate of 8010 ± 25 ^14^C B.P., or 8.99–8.65 cal. ka (Supplementary Information: WMP 1 radiocarbon data). This estimate is nearly identical to that of the WMP 6 estimate of 9.00–8.65 cal. ka^12,33^, indicating that the two burials were contemporaneous or nearly so.

## Discussion

The consistent finding of both projectile and knife wear on the Wilamaya Patjxa points indicates that the tools served both projectile and cutting purposes for the female and male individuals who lived roughly at the same time 9000 years ago in the high Andes. The observations furthermore exclude strict versions of the knife and offering models. How do we then interpret the association of projectile points with the two individuals? Coupling these findings with the widespread cultural regularity of associating funerary objects with gender and labor identities of deceased individuals^34^^22^, we conclude that the female and male individuals at Wilamaya Patjxa were likely large-mammal hunters.

It is important to recognize that this analysis does not entirely exclude a more complicated grave-offering model in which living community members donated their used tools to the deceased as a symbolic gesture^2^. We could suppose that males were buried with the tools they used in life and females were buried with tools that their male counter-parts used in life but that the females themselves did not use. However, such a model is inconsistent with ethnographic observations on tool-gender-labor associations^34^. As a matter of analytical parsimony, we consider such complicated offering models unlikely to account for the observations.

That large-mammal hunting may have been a gender-neutral activity in the Andean highlands 9000 years ago, and ostensibly elsewhere in the early Americas^12^, raises the question of why the archaeological pattern differs from the ethnographic pattern where large-mammal hunting is the purview of males. A recent experimental study shows that atlatl technology may encourage broad participation in hunting activities^3^. Forager ethnography furthermore identifies two important socioecological contexts that would have favored widespread participation in large-mammal hunting. First, communal hunting entails increasing returns to scale that encourage joining and recruitment of participants to a point^35–37^. Given that the earliest forager populations were small, all able-bodied community members would have had incentive to participate in communal hunts. Moreover, communal hunting reduces some impediments of child care on hunting. It does not require stealth, obviating concerns about noisy children scaring target animals, and children can directly contribute to the effort by driving animals. Indeed, females and children among the Aka of central Africa routinely participate in group net hunts for duikers^9^.

A second ecological context that favors female hunters is that in which campsites are proximate to hunting grounds. Such geographic proximity makes alloparenting more accessible, freeing breast-feeding females to pursue solo or small-group hunts. Agta females in the Philippines are observed to routinely hunt large mammals singly or in small groups because ecological circumstance places the hunting grounds close to camp^10^. Although the Agta are a rare exception to the man-the-hunter rule among ethnographic foragers, their ecological circumstance may have been the rule for early forager societies whose residential mobility patterns would have put camps in close proximity to prey^38^.

Whether atlatl technology, communal hunting, or individual hunting applied to the Wilamaya Patjxa case requires further investigation. Atlatl technology was likely the major hunting weapon of Holocene foragers of the Andean highlands as suggested by atlatl parts, projectile point morphology, and rock art imagery in the region or adjacent regions^13–15,39,40^. However, direct evidence of atlatl technology from early Holocene contexts remains elusive. Although communal hunting architecture is evident in many other regions around the world^41^, it is currently unknown in the region surrounding Wilamaya Patjxa, despite intensive archaeological survey^42–44^, provisionally suggesting that individual or small-group hunting strategies were at play.

The extent to which labor practice was gendered among early human societies remains an active area of research. Although it is impossible to directly observe those practices, ethnographic and archaeological records offer key insights for evaluating competing hypotheses. By examining usewear patterns on stone tools associated with female and male burials at Wilamaya Patjxa, this study eliminates the possibilities that the tools were strictly knives or grave offerings. Rather, the results show that the points were used as both projectiles and knives, consistent with the hypothesis that females and males at Wilamaya Patjxa participated in large mammal hunting. This finding is furthermore consistent with ecological models that posit Late Pleistocene and Early Holocene conditions to have favored broad participation in hunting activities.

## Materials and methods

Lithic usewear analysis was performed on 18 artifacts from the Andean highland site of Wilamaya Patjxa, 9 cal. ka. In addition, radiocarbon dating of bone collagen from one individual—WMP 1—was conducted. Here, we describe our analytical materials and methods.

### Wilamaya Patjxa

The archaeological site of Wilamaya Patjxa is located at 16.2° south latitude, 69.7° west longitude, 3925 m in elevation in the Puno district of southern Peru (1). The site covers approximately 1.6 ha and consists of a moderate-density surface scatter of lithic artifacts that have been disturbed by agricultural plowing to 30 cm below the surface. The geologic surface on which the site lies appears to have been stable throughout the Holocene^45^ resulting in a palimpsest occupation surface that has been mixed by plowing. Despite this site disturbance, intact or partially intact cultural pit features extend below the plow zone and thus offer an opportunity to examine discrete behavioral activities with a high degree of temporal control. Excavations in 2018 revealed a series of such features including five human burial pits containing a total of six individuals. Two of the individuals were interred with lithic tools, which are the subject of this analysis.

Excavation of the individuals was performed with wood spatulas, bamboo skewers, brushes, and compressed air via aspirator bulbs to minimize damage and abrasion to the skeletal materials and artifacts. Dirt was mechanically removed from artifacts using brushes, and all artifacts were curated in 4 mil acid free plastic bags with paper tags. Each artifact was curated in its own bag except in the case of artifacts 941a—d, which were curated in the same bag.

### Wilamaya Patjxa Individual 1

Wilamaya Patjxa Individual 1 (WMP 1) is an adult male interred in a shallow pit extending 9 cm below the plow zone, approximately 40 cm below ground surface. Osteological remains consist primarily of fragmentary cranial bones and postcranial long bones. The individual was interred in a flexed position on their left side with head oriented east. They experienced heavy tooth wear, antemortem tooth loss of a right mandibular molar, and a carious lesion with infection in a left mandibular molar. A previous attempt at direct dating using an ultrafiltered sample of collagen from the right petrous portion was unsuccessful^12^.

Two flaked stone artifacts were recovered in situ in direct association with WMP 1. A fine-grained volcanic bi-point form identified as 3E style—a Middle Archaic Period form dating to 9.0–7.0 cal. ka^46^—was located in the pelvic area. The bone was highly degraded occluding topological detail about the association between the point and the individual—i.e., whether it was embedded in bone.

A white chert or chalcedony stemmed form was located under and in contact with the proximal end of the right radius or ulna. Again, the skeletal elements were highly degraded preventing any further details about the topological association between the point and the individual. Prominent denticulates occur along the blade margins. The artifact is missing the distal-most and proximal-most portions, and macro-scale abrasion is evident on the haft element, all suggesting use prior to burial. It is identified as a 3B form, again a Middle Archaic Period form dating to 9.0–7.0 cal. ka^46^.

### Wilamaya Patjxa Individual 6

Wilamaya Patjxa Individual 6 (WMP 6) is a young adult female interred in a burial pit near the center of the site and extending 55 cm below the agricultural plow zone, approximately 85 cm below the ground surface. Preservation of the osteological materials is poor, consisting of fragmentary cranial and longbone elements. Osteological and amelogenin protein analysis of the well-preserved teeth secure a female sex estimation and establish that the individual was 17–19 years old at the time of death. The individual was interred in a semiflexed position on their left side in direct stratigraphic association with 24 stone artifacts, all in situ^12^.

Six eared projectile points of 1B style indicative of an Early Archaic Period burial date to sometime between 11 and 9 cal. ka^46^. Two radiocarbon dates taken on bone collagen average to 8008 ± 16 ^14^C before the present (B.P.), or 9.00–8.65 cal. ka^12,33^. Isotope bone chemistry further indicates that the individual occupied the highlands on a permanent basis and that they consumed a mixed diet of terrestrial plants and animals. Large-mammal bone, including the lumbar vertebra of a taruca (*Hippocamelus antisensis*) or Andean deer, in the feature fill further attests to the importance of large-mammal hunting.

Twenty of the 24 associated artifacts were tightly concentrated and partially stacked in a pile just above the femora. The stacking and topological integrity of the artifact cluster indicate that the artifacts were likely interred as an integrated toolkit in a perishable container such as a leather bag. At the base of the artifact stack was a large igneous river cobble with a unidirectionally flaked working edge at one end and a rounded pestle-like surface on the opposite end. Piled on the cobble were four complete 1B-style chert projectile points, two chert thumbnail end scrapers, two large igneous scrapers/choppers, a possible backed knife, two retouched chert flakes, three unmodified chert lithic flakes, and a red ocher nodule. Adjacent to and in contact with the artifact stack were two small, well-rounded river cobbles and two red ocher nodules. The large river cobble and one of the small cobbles show ocher staining on the acute ends. In addition to the artifact cluster, isolated artifacts were found on the burial pit floor including a complete 1B-style projectile point, projectile point midsection, projectile point tip, and retouched laminar flake. It is unclear if the displacement of these artifacts relative to the cluster was systemic or post depositional.

Based purely on morphology alone, the projectile points were presumably used for dispatching large mammals, the backed knife and lithic flakes to field dress them, and the small scrapers for detailed hide work. The function of the large scrapers/choppers is unclear but may have been used to extract bone marrow or process hides. The flaked stone portion of the kit is notably similar to the optimal mobile toolkit theorized by Kuhn^47^. The spatial co-occurrence of the flaked stone tools with red ocher and ocher-stained cobbles suggest that the ocher and cobbles were related to animal-hide processing^48^.

Macroscopically visible breakage and wear patterns indicate that at least some artifacts in the assemblage were used prior to interment^12^. Artifact 5, a projectile point, shows retouch along one margin, and artifact 6 is a projectile point mid-section, suggesting breakage during use. One end of artifact 17, a large multi-tool, shows evidence of battering, and one of the lateral margins exhibits macroscopically visible ocher staining and striations oriented with the tool’s length, suggestive of polishing or burnishing a hard surface. Artifact 19, which is a large chopper or scraper tool made from a dense ferric material, exhibits extensive wear along one of the flaked working margins suggesting prolonged and intensive use. Three flakes (artifacts 11–12 and 14) in the artifact stack exhibit worked edges suggesting possible use. Artifacts 8–10 exhibit minute traces of what could be edge modification due to use.

### Usewear analysis

Although the aforementioned observations reveal macro-scale indications of artifact use, until this analysis, the artifacts had not been subjected to formal usewear analysis. Prior to usewear analysis, lithic artifacts were cleaned with water and ethanol to remove residues^49^. All specimens were examined on a Leica DM750 microscope at magnifications of 40x, 100x, 200x, and 500x. Geologic samples representing the raw materials in the Wilamaya Patjxa assemblage were microscopically examined to document the natural characteristics of the materials in their unused state.

The locations of macro- and micro-flaking patterns and microscopic traces, such as polish and linear indicators, were documented for each artifact. An MC170 HD digital camera was used to acquire usewear images using Leica LAS X software including the Image Builder Z package. Polishing refers to shape altering and smoothing of the stone surface through the combination of tensile stress and the mechanical removal of asperities on the microtopography^50^. This process changes the reflectivity of the surface, making the polished location brighter than other surfaces on the stone. For this study, polish development was recorded based on both the general distribution of wear on the entire tool and the degree of wear intensity at specific locations on the tool surface. Linear indicators refer to microscopic evidence of motion on a tool’s surface. These can be in the form of polish streaks, polish distributed linearly, or striations, furrows, or grooves on a tool’s surface caused by use^50^. Linear indicators are used to understand tool use-trajectory and to infer function^28^. For this study, both the type of linear indicator and orientation were recorded. Ultimately, the spatial distribution and intensity of usewear (i.e., polish and linear indicators) are used to infer tool use.

### Experimental controls

We reference the series of actualistic experiments and resulting collection of usewear images developed by Smallwood and colleagues, which are summarized as follows: replica bifacial points were hafted on spears and thrusted in animal carcasses, documenting the accrual of traces through a series of uses^51–53^; bifacial serrated and serrated-beveled points were hafted on darts and launched with atlatls at carcasses^54^; hafted bifacial unserrated and serrated points were used to butcher animal carcasses, slice meat, and cut raw hide^52,54^; bifacial beveled points were used as perforators to punch leather^54^; bifacial unserrated and serrated points were used as multi-functional tools through documented series of uses, performing as both projectiles and knives; and bifaces were used to chop and scrape wood^52^.

All experimental tools were photographed before, during, and after the trials. Tool use, performance, and changes were recorded, as well as the properties of contact materials. Experimental tools were analyzed after each series of use-episodes to monitor the rate and intensity of usewear accrual. Table S1 summarizes the major findings of each experiment. These observations are in agreement with other experimental usewear analyses^55,56^.

Based on the results of these experiments, we apply the following observations to examine the ostensible projectile points associated with both female and male individuals at Wilamaya Patjxa. Projectile points that have experienced high-velocity impacts, especially when contacting bone or other hard surfaces, tend to acquire microscopic traces on high, distal-facing arises and facets of the microtopography at the point’s tip, extending proximally along the center axis of both faces. Further, linear indicators, in the form of striations and polish, are oriented parallel to the long-axis in the direction of impact and penetration. In contrast, knife wear tends to leave traces along the blade margins on both the distal and proximal aspects of flake scars, denticulates or serrations, and in negative flake scars and other low areas of a working edge’s microtopography. Linear indicators initiate at and are oriented perpendicular or oblique to the blade margin where butchering and initial cutting takes place and parallel with the slicing-portion of the blade^52,54^.

Because much of Smallwood’s experimental program has focused on bifacial point functionality, we also reference the experimental work of other usewear analysts as analogues for the detection and explanation of usewear traces on the artifacts from Wilamaya Patjxa^26,30,31,57,58^.

### Laser scanning

In order to document and visualize usewear patterns, 3D shaded relief models were derived from laser scans of each artifact. An EinScan Pro 2X was used to capture 3D point clouds. Mesh models were created with the accompanying ExScan Pro v. 3.7.3.0 software package. Models were manipulated in MeshLab v. 2020.07, and the Z-painting tool was used to color-shade artifact surfaces with documented usewear.

### Radiocarbon dating

A previous radiocarbon dating effort successfully produced two radiocarbon dates from the bone of WMP 6, which are summarized above. However, the previous attempt at collagen extraction for WMP 1, the male individual, was unsuccessful at that time. We therefore made a second attempt at extraction.

Collagen extraction and stable isotope measurements were carried out at the Keck Carbon Cycle AMS laboratory at University of California, Irvine. A small bone fragment was taken from the left petrous portion of Individual 1. Bone sample preparation followed^59^. Bones were cleaned mechanically with a Dremel rotary tool, and aliquots of crushed bone (~ 200 mg) were decalcified in 1N HCl at room temperature overnight, gelatinized at 60 °C and pH 2, and ultrafiltered to select a high molecular weight fraction (> 30 kDa), which was freeze-dried. Aliquots for ^14^C were combusted in vacuo in quartz at 900 °C with CuO, graphitized, and measured by AMS.

Radiocarbon results are given as conventional radiocarbon ages following the conventions of Stuiver and Polach^60^. Sample preparation backgrounds have been subtracted based on measurements of ^14^C-free collagen, and all results have been corrected for isotopic fractionation using ^δ13^C values measured on prepared graphite using the AMS spectrometer. The ^δ13^C and ^δ15^N values shown here were measured to a precision of < 0.1 h and < 0.2 h, respectively, on aliquots of ultrafiltered collagen, using a Fisons NA1500NC elemental analyzer/Finnigan Delta Plus isotope ratio mass spectrometer.

Calibration of the δ^13^C value was performed with the 2020 Southern Hemisphere calibration curve^33^ as implemented with Bchron^61^ in R statistical computing environment^62^. Calibrated age estimates are presented as 95% confidence ranges.

### Supplementary Information

Supplementary Information 1.

## Data Availability

All data required to reproduce these results are presented in the main text and Supplementary Information.
