# Supplementary material for: Lithic usewear confirms the function of Wilamaya Patjxa projectile points
Source: Sci Rep. 2023 Nov 3;13:19044. doi: 10.1038/s41598-023-45743-7 (PMC10624878; doi:10.1038/s41598-023-45743-7)
Supplement: Supplementary file 1 — Supplementary Information 1. [file 41598_2023_45743_MOESM1_ESM.pdf]

Supplementary Information

Lithic usewear confirms the function of Wilamaya Patjxa projectile points

Ashley Smallwood[1]*, Randall Haas[2]*, Thomas Jennings[1]
[1]University of Louisville, [2]University of Wyoming
*corresponding authors, a.smallwood@louisville.edu, whaas@uwyo.edu

Contents

Table S1: Usewear control experiments referenced in this analysis....2
Usewear data....3
- WMP1-Artifact 25....3
- WMP1-Artifact 26....3
- WMP6-Artifact 1....3
- WMP6-Artifact 2....4
- WMP6-Artifact 3....4
- WMP6-Artifact 4....5
- WMP6-Artifact 5....5
- WMP6-Artifact 6....5
- WMP6-Artifact 7....6
- WMP6-Artifact 8....6
- WMP6-Artifact 9....6
- WMP6-Artifact 10....6
- WMP6-Artifact 11....6
- WMP6-Artifact 12....7
- WMP6-Artifact 13....7
- WMP6-Artifact 14....7
- WMP6-Artifact 15....8
- WMP6-Artifact 16....8
- Unexamined artifacts....8

WMP 1 radiocarbon data....9

Table S1: Usewear control experiments referenced in this analysis.

Use activity	Location of wear and distribution of wear traces	Orientation of linear indicators	Refs.
Penetration/ Piercing with Impact of Bifacial Points	Polish elements concentrated on high areas of the microtopography and flake scar arrises and distal-facing facets at the point's tip and extended proximally along the center-axis; contact with hard material (e.g., bone) can produce microflaking at point tip, including snap fractures and impact burins<br><br>If point is beveled, traces were acquired on the high medial plane between the bevel ridges from the tip to just above the haft and on the flake scar ridge created by the bevel.<br><br>Usewear evident on dorsal and ventral surfaces	Parallel to the long-axis in the direction of impact and penetration	[1, 2]
Cutting/ Butchering with Bifacial Points	Polish distributed in isolated use-areas along the lateral edge; polished surfaces on proximal-facing facets along the lateral edge; contact with hard worked material (e.g., bone) can cause microflaking on and perpendicularly removed from working edge<br><br>If serrated edge, polish interrupted by serrations; polished surfaces on apex and proximal-facing facets of individual serrations; polish down in negative flake scars between serrations along working edge<br><br>For bifaces potential for differential wear on one or both laterals depending on working edge preference, differential wear on dorsal/ventral faces determined by greater pressure placed on one face.	Oblique to perpendicular linear indicators initiating at the cutting edge	[1, 2]
Perforating/ Punching with Bifacial Points	When tool is beveled, polish acquired at the point tip, along the ridge formed by the point's bevel, the lateral edge opposite the beveled edge, and inside flake scars that created the bevel; point tip was slightly rounded and polished.	Along beveled ridge, striations oriented perpendicular to the long-axis, a pattern created by the twisting motion.	[2]
Chopping with Bifacial Tools	When tool is a bi-convex bifacial edge, polish acquired in isolated units and high areas of microtopography along working edge. Microflaking on working edge including step-fractures and hang flakes<br><br>Usewear evident on dorsal and ventral surfaces of working edge, but differentially distributed depending on angle hafted/held during use	Linear indicators oriented perpendicular to the working edge.	[1]
Scraping with Bifacial Tools	When tool is a bi-convex bifacial edge, polish occurs directly on working edge<br><br>Usewear differentially distributed depending on the motion of use; toward the worker distributes more usewear on the dorsal, and away from the worker distributes usewear on the ventral faces	Linear indicators oriented perpendicular to the working edge.	[1]

Usewear data

Here we present the observational details for usewear analysis of two artifacts associated with WMP1 male individual and 16 artifacts associated with WMP6 female individual. Artifacts are identified by figure numbers as presented in Fig. 1 and Haas et al. [3]. Supplementary Figs. S1-18 illustrate macroscopic images of the artifacts, examples of microscopic images captured, and the specific locations of those images (e.g., Fig. 3). When available, these figures also include 3D models of the artifacts depicting the artifact-wide distribution of usewear traces.

WMP1-Artifact 25

Artifact 25, a nearly complete 3B-style white chert or chalcedony point recovered from under and in contact with WMP1's right radius or ulna, was used as a projectile and knife. This point has fine serrations along both blade margins from the tip to the point's shoulders.

Artifact 25 acquired polish from use as a projectile distributed from the points' broken tip proximally along the center axis (Fig. S1.1). Usewear traces along the blade margins indicate this point was also used as a knife. Polish occurs on isolated facets along the blade margins on flake scar arrises and down in negative flake scars between serrations. One blade margin acquired scalar microflaking from use, and polish occurs within that damage (Fig. S1.2). Serrations on the blade margins were polished on the apex, distal-facing, and proximal-facing surfaces (Figs. S1.3 and S1.5). The left blade margin acquired extensive polish, particularly on proximal-facing facets of prominent serrations in the medial portion of the point (Fig. S1.4).

WMP1-Artifact 26

Artifact 26, a nearly complete fine-grained volcanic 3E style bi-point form recovered from WMP1's pelvic area, was used as a projectile. Both the dorsal and ventral faces acquired polish from the distal to the medial axis of the point. Collateral flaking created a prominent ridge on the point's long-axis, and through impact, polish was acquired along this center axis on high facets on the microtopography and distal-facing facets of flake scars (Fig. S2.1).

WMP6-Artifact 1

Artifact 1, a complete 1B-style chert point recovered from the stack of artifacts associated with WMP6, was used as a projectile and knife. The point's tip and center axis, on both the dorsal and ventral faces, have multiple usewear traces indicative of use as a projectile. The tip has a snap break likely acquired from impact. Polished facets are evident on high medial ridges and distal-facing facets along the center axis from the point's tip to below the ears, in some cases creating rounded arrises that indicate contact with hard material (Fig. S3.1). Striations occur along the center axis and run parallel to the long axis of the point following the direction of motion (Fig. S3.2); on one point face, striations on a high medial ridge measured 200 μm in length (Fig. S3.3).

Both lateral blade margins have evidence of use acquired from cutting, and interestingly, the point's ears acquired usewear, suggesting this attribute was a part of the functional unit. One lateral margin has minimal evidence of use, scalar microflaking and light polish occur along the blade margin. The opposite lateral blade margin has comparatively more evidence of use. This margin also has microflaking from use—notably an isolated portion along this margin has a series of flake scars with stepped terminations, suggesting this segment of the blade contacted

hard material (Fig. S3.4). Polish is localized to the edge but occurs within negative flake scars and on proximal-facing facets of flake scar arrises along the blade (Fig. S3.5). Along the same lateral margin, the ear has polish on distal- and proximal-facing surfaces. On the proximal-facing surface of the ear, a series of striae are oriented parallel to the blade margin, again indicating the point ear was a portion of the functional unit and the back of the ear came in contact with worked material (Fig. S3.6). Below the ears, in the point's stem, isolated polish occurs on proximal-facing facets. These usewear traces are indicative of wear produced from hafting.

WMP6-Artifact 2

Artifact 2, a complete 1B-style chert point recovered from the stack of artifacts associated with WMP6, was used as a projectile and knife. Evidence of use as a projectile was acquired on the dorsal and ventral faces along the center axis from the point's tip to just below the ears. This point has a medial ridge formed by the intersection of flake scar terminations, and in this area along the center axis, high surfaces on the microtopography and distal-facing facets of arrises were highly polished from penetration and contact with hard material (Fig. S4.1). Parallel striae show the direction of motion.

Artifact 2 was also used for cutting, and the evidence was acquired on the blade edge and the protruding point ears. Both lateral edges have isolated evidence of cutting, but the right margin (Fig. S4.2 and S4.3) acquired to most evidence of use, including striae oriented parallel to oblique to the cutting edge. Point ears were highly polished (see Fig. S4.2) and rounded on the distal and proximal-facing surfaces (see Fig. S4.3). One ear has evidence of microflaking on the proximal-facing surface (Fig. S4.4). Evidence on the point's ears suggests these attributes were in regular contact with worked material, and in some tasks, that material was hard enough to create microflaking and rounding.

WMP6-Artifact 3

Artifact 3, a complete 1B-style chalcedony point recovered from the stack of artifacts associated with WMP6, was used as a projectile and knife. This point acquired polish from the broken tip extending proximally along the center axis on both the dorsal and ventral faces. Flake scar terminations of parallel collateral flaking created high facets on the microtopography that acquired the most intensive polishing (Fig. S5.1). Striae located along and oriented parallel to the center axis show the direction of projectile motion (Fig. S5.2). The distribution of polish and stria, located as far as 0.5 mm below the proximal-most lateral ear, indicate the hafting mechanism began below the lateral ears [4].

Artifact 3 has one blade margin with two isolated ears or serrations, and this blade margin acquired evidence of cutting. Polished facets along the blade margin occur on high flake scar arrises and down in negative flake scars (Fig. S5.3). Striae localized to the blade are oriented oblique to the working edge. Usewear traces indicate both ears were in contact with worked material during use, confirming the features were a part of the working margin rather than bound in the haft. On the protruding point ears, the apex (Fig. S5.4), distal facing, and proximal facing facets are polished. The high surface created by the bevel above the lateral ear acquired striae oriented oblique to the blade margin, suggesting the degree of penetration along the blade edge (Fig. S5.5 and S5.6). The portion of the blade edge between the ears also acquired polish but no striae (Fig. S5.7).

WMP6-Artifact 4

Artifact 4, a complete 1B-style chert point recovered from the stack of artifacts associated with WMP6, acquired only minimal evidence of use as a projectile. A medial ridge along the center axis, created by terminations of parallel collateral flaking, acquired polish on the highest facets of the microtopography (Fig. S6.1). Lateral edges and lateral ears have no evidence of polish (Fig. S6.2).

WMP6-Artifact 5

Artifact 5, a complete 1B-style chert point recovered near the stack of artifacts associated with WMP6, was used as a projectile and knife. This point acquired projectile wear polish from the broken tip extending proximally along the center axis on both the dorsal and ventral faces. Along the center axis, surfaces located on a high medial ridge formed by terminations of flake scars and distal-facing facets of those arrises are polished—indicating the direction of impact (Figs. S7.1 and S7.2). Along the center axis, where the point's cross-section is the thickest, striae are oriented parallel to the long-axis (Fig. S7.3).

Artifact 5 has one lateral margin with two prominent, isolated ears, and this blade edge has evidence of use. Polish is restricted to directly along the blade edge and occurs in low surfaces of the microtopography, between small serrations and down in negative flake scar (Fig. S7.4). Both ears have polish on the apex (Fig. S7.5), distal facing, and proximal facing facets are polished. The proximal-most ear on this blade margin has polished facets on the proximal face of the ear and a series of short striae oriented oblique to the lateral edge (Fig. S7.6), suggesting the back of the ear contacted worked material in a cutting motion.

WMP6-Artifact 6

Artifact 6 is an incomplete 1A or 1B-style chert point midsection recovered near the stack of artifacts associated with WMP6. The point has a hinge fracture at the point distal and snap break at the base. Artifact 6 has a complex use-life with evidence of use as projectile and knife prior to the breaks and usewear evidence on the breaks that suggest the point was recycled as a scraper.

Along the center axis, on a high surface created by collateral flaking with stepped terminations, a series of striae are oriented parallel to the long axis. These linear indicators and additional polish along the center axis indicate the direction of motion with use as projectile (Fig. S8.1).

Blade margins and ears of the point midsection acquired evidence of use as knife. Polish occurs along the sinuous edge of the lateral blade margin (Fig. S8.2). Ears are highly polished, with linked polish on surfaces of the apex and proximal-facing facets on both point faces (Fig. S8.3). One point ear has linear indicators in the form of polish oriented from the apex of the ear inward toward the center axis and a striae oriented oblique to the blade margin (Fig. S8.4).

Interestingly, usewear on the hinge and snap breaks of the midsection suggest this point was recycled and used after breaking. Based on the extent of linked polished facets on the point's distal break, this edge contacted hard material that smoothed the surface of the edge and margin face (Fig. S8.5). The presence of striae located on and oriented oblique and perpendicular to this broken edge indicates that the distal break of the point midsection was used in scraping or whittling tasks (Fig. S8.6). On the reverse face of the point, the edge created from the proximal snap fracture, also acquired usewear traces in the form of polish and a series of perpendicular

stria. Polish with pitting on this surface suggests extensive contact with hard material, such as bone, likely worked with a scraping or planning motion (Fig. S8.7) [5, 6]. The resourceful recycling explains why the fragmented point was not discarded and remained an active component of WMP6's toolkit until her death.

WMP6-Artifact 7

Artifact 7 is a point tip found on the burial pit floor associated with WMP6. The tip was broken with a snap break and acquired evidence of use as a projectile. The point fragment acquired polish from the snap break at the distal-most portion of the tip (Fig. S9.1) along the center-axis to the proximal break on both the dorsal and ventral faces. Polish occurs on high surfaces of the microtopography and distal-facing facets (Figs. S9.2, S9.3, and S9.4).

WMP6-Artifact 8

Artifact 8 is a chert flake recovered in the stack of artifacts associated with WMP6. Macroscopic edge modification from use is minute, and microscopic evidence is also minimal. Usewear traces are isolated to a functional unit less than 1 cm in length along a concave edge of the flake margin. Polish was acquired on low and high surfaces in this portion of the flake, suggesting contact with soft material (Fig. S10). The morphology of the flake, the restricted extent of the usewear in the concavity, and the distribution of polish on the microtopography suggest this use unit was used for scraping.

WMP6-Artifact 9

Artifact 9 is a chert flake recovered in the stack of artifacts associated with WMP6. This flake acquired no evidence of use. Post-excavation, this artifact was curated with another artifact in the same plastic bag, causing flake-on-flake damage that is microscopically detected and clearly differentiated from usewear based on the location, appearance, and unpatterned nature of the traces (Fig. S11) (see [7]).

WMP6-Artifact 10

Artifact 10 is a chert flake recovered in the stack of artifacts associated with WMP6. This artifact acquired no evidence of use (Fig. S12).

WMP6-Artifact 11

Artifact 11 is a retouched chert flake recovered in the stack of artifacts associated with WMP6. This modified flake tool has bimarginal retouch and usewear traces indicating there were two functional units—one on the dorsal surface convex margin and one on the ventral surface concave lateral near the flake distal.

The convex edge on the dorsal flake surface has macroscopically-visual edge modification and microflaking with a series of stepped terminations. This portion of the flake margin acquired some rounding and polish oriented perpendicular to the edge and limited to high flake arrises off that margin (Figs. S13.1 and S13.2). The functional unit on the ventral surface is limited to a smaller portion of the flake margin. Macroscopic edge modification and microflaking scars are isolated along the margin of the flake distal, and only high arrises have

been polished from contact material. Artifact 11 is a modified flake tool used in scraping/whittling tasks.

WMP6-Artifact 12

Artifact 12 is a retouched chert flake recovered in the stack of artifacts associated with WMP6. This flake tool has evidence of use located on two lateral margins at distal portion of the flake. The greatest evidence of use occurs on the straight to convex lateral of the dorsal surface, where there is macroscopic chipping with polish in negative flake scars and on high arrises of the modified flake margin (Figs. S14.1 and S14.2). On the opposite lateral margin near the distal of the dorsal surface, the flake acquired some evidence of use in the form of polished facets restricted to the otherwise unmodified edge (Fig. S14.3). Usewear traces were restricted to these functional units directly on the flake tool edges with no evidence of usewear on the interior portions of the flake (Fig. S14.4). While a lack of linear indicators make determining the direction of motion difficult, usewear suggests this flake tool was used minimally in cutting tasks on hard and soft materials.

WMP6-Artifact 13

Artifact 13 is a retouched chert flake recovered in pit floor near WMP6. The morphology of the flake, with a triangular planview and thick triangular cross-section at the distal portion, suggests it could be scraper. Unfortunately, directly analyzing the surface of the thickest end of the flake blank, or potential bit, was not possible due to a lack of vertical relief between the microscope stage and objectives. However, usewear traces on one lateral margin suggest the flake was utilized. The retouched convex lateral margins has polish on flake scar arrises along the sinuous edge, suggesting the edge contacted hard material that smoothed the highest points on the microtography (Figs. S15.1 and S15.2). The distal-most point of this margin, where the lateral margin meets the bit edge, is also highly polished (Fig. S15.3). While this flake tool does not have the extent of macroscopic retouch and usewear traces of the curated endscrapers in this assemblage, the polished margins suggest utilization as a scraper.

WMP6-Artifact 14

Artifact 14 is a retouched chert flake recovered in the stack of artifacts associated with WMP6. The morphology of this modified flake suggests it could have been side scraper, but the retouch patterns and usewear traces indicate it functioned as a backed knife. The concave lateral flake margin has retouch and scalar flaking but no evidence of microflaking with stepped terminations. This margin did acquire usewear polish on both the dorsal and ventral surfaces directly along the edge. On the ventral surface of this edge, polish was acquired in low and high place locations of the microtopography, and on the dorsal surface, only ridges formed by flake scar arrises are polished (Figs. S16.1–4). This differential distribution, suggests the tool was held at an angle other than 90 degree and used in a slicing or carving motion [8]. Further, striae slightly oblique to the lateral edge near at the proximal-most portion of this margin suggest this portion of the margin was used to make initial cuts in the carving process (Fig. S16.5).

WMP6-Artifact 15

Artifact 15 is a retouched chalcedony flake recovered in the stack of artifacts associated with WMP6. The flake was modified as a triangular, or thumbnail, endscraper, with the convex working edge on the proximal portion of the flake blank. Usewear traces confirm Artifact 15 was utilized in scraping tasks. Unfortunately, directly analyzing the dorsal surface of the thickest end of the flake blank, or bit, was not possible due to a lack of vertical relief between the microscope stage and objectives. The ventral surface of the bit acquired extensive evidence of use. This edge was rounded with polish, microchipping, and pitting (Figs. S17.1 and 2)—a pattern indicating the scraper contacted material harder than fresh meat or soft wood and more similar to dry hide [8, 9, 10, 11]. Also on the ventral surface, a series of striae oriented oblique to the edge demonstrate the direction of motion (Figs. S17.3 and 4).

The polish development and distribution are similar to polish characteristics found on experimentally and ethnographically utilized and documented endscrapers [10, 11, 12, 13]. Takase [14] demonstrated that endscrapers used in a pushing motion acquired the wear traces on the ventral/interior contact surface rather than the dorsal surface (also see [15]). While the dorsal surface could not be analyzed, based on the observed usewear traces on the ventral surface and edge, the endscraper could have been used in a scraping task that required pushing the ventral/interior surface of the scraper bit against the worked material—potentially buffing or membraning dry hides [16].

WMP6-Artifact 16

Artifact 16 is a retouched chert flake recovered in the stack of artifacts associated with WMP6. The flake was modified as a triangular, or thumbnail, endscraper with the convex working edge on the proximal portion of the flake blank. Usewear traces confirm Artifact 16 was utilized in scraping tasks. The working edge was rounded with polish, microchipping, and pitting (Fig. S18.1). Usewear traces also roll over onto the dorsal surface on the bit of the tool. The most extensively polished facets occur in the center of the bit, on the edge's two lower-most tiers of scalar flaking (Figs. S18.2 and 3), but traces also extend approximately 0.25 cm away from the convex working edge, spanning the extent of retouch flake scars (Fig. S18.4). Usewear traces, comparatively minimal, were acquired on the ventral surface of the convex edge in the form of polish localized to the edge margin and oriented perpendicular to the edge, as well as isolated microchipping (Figs. S18.5 and 6).

The location of usewear traces suggests the endscraper was used in a pulling motion, during which the dorsal surface came in contact with the worked material [14], and rounding and microchipping suggest the contact material was hard, producing traces similar to experimental scraping of dry hide [10].

Unexamined artifacts

Unfortunately, artifacts 17, 18, 19, 20, and 21 were not analyzed for usewear due to their large size, which could not be accommodated with the microscope stage relief used for this analysis. Based on macroscopically visible edge modification, future usewear analyses could prove productive in determining how these artifacts were used.

WMP 1 radiocarbon data

The analytical and quality-control results for the WMP 1 sample are as follows (Fig. S19):

- sample ID: UCIAMS 259854
- sample material: left petrous portion of WMP 1
- $\delta^{13}C$: -18.4±0.1 ‰
- fraction modern: 0.3689±0.0009
- $\delta^{14}C$: -631.1±0.9 ‰
- ^{14}C age: 8010±25 BP
- >30kDa collagen yield: 5.6 %
- $\delta^{15}N$: 8.6 ‰
- $\delta^{13}C$: -18.4 ‰
- %N: 16.0
- %C: 44.1
- C/N (wt%/wt%): 2.8
- C/N (atomic): 3.3

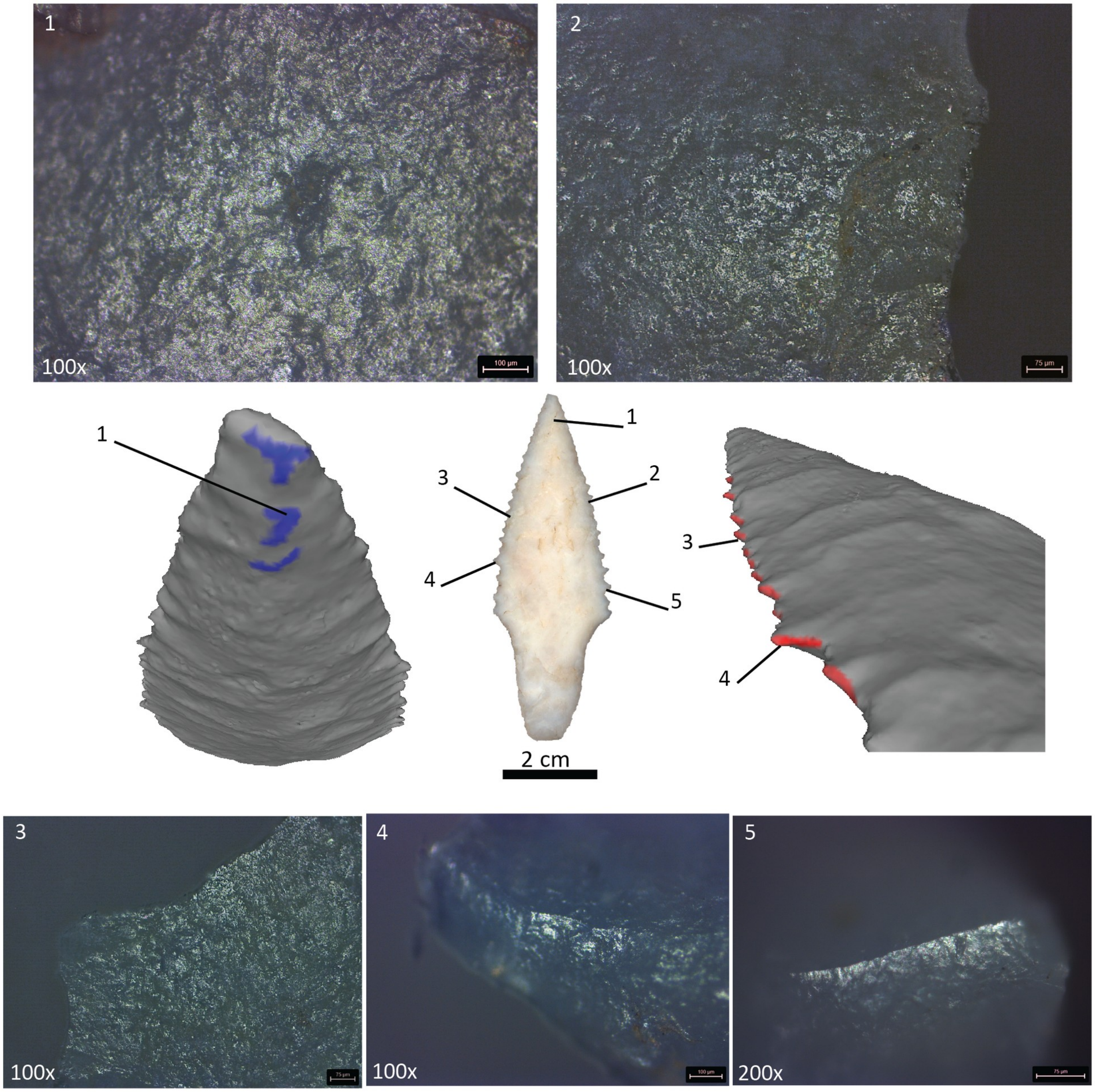

Fig. S1: WMP1-Artifact 25 acquired evidence of use as a projectile and knife. *1) Polish from use as projectile on a high surface at the point's center axis. 2) Scalar flaking and polish on blade margin from use as a knife. 3–5) Serrations on blade margins with polish on the apex, distal-facing, and proximal-facing surfaces from use as a knife.*

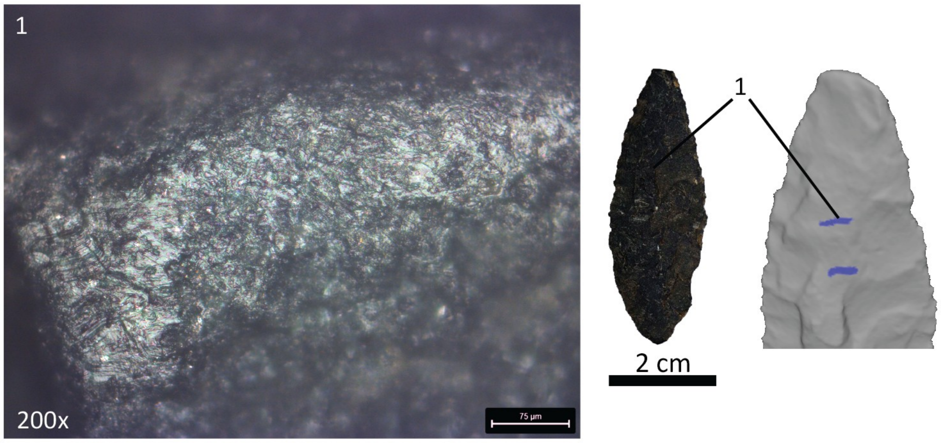

Fig. S2: WMP1-Artifact 26 acquired evidence of use as a projectile. *1) Polish on a high, distal-facing facet along the point's center axis.*

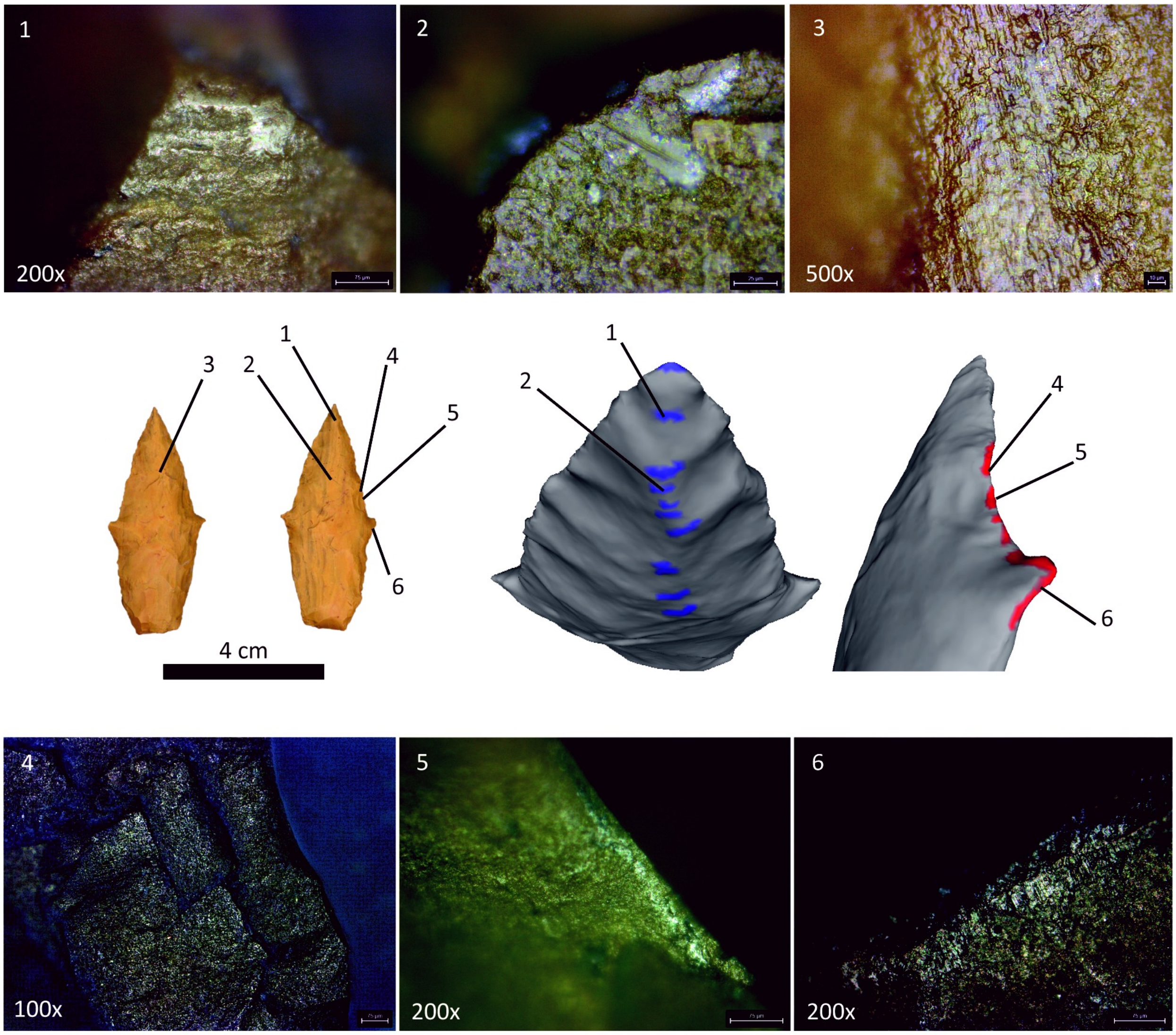

Fig. S3: WMP6-Artifact 1 acquired evidence of use as a projectile and knife. *1) Polish from use as a projectile occurs on the arrises of both faces. 2, 3) Striations oriented parallel to long-axis in direction of impact. 4, 5) Microflaking and polish on blade margins from use as a knife. 6) Striations on the proximal aspects point ears, indicating knife use.*

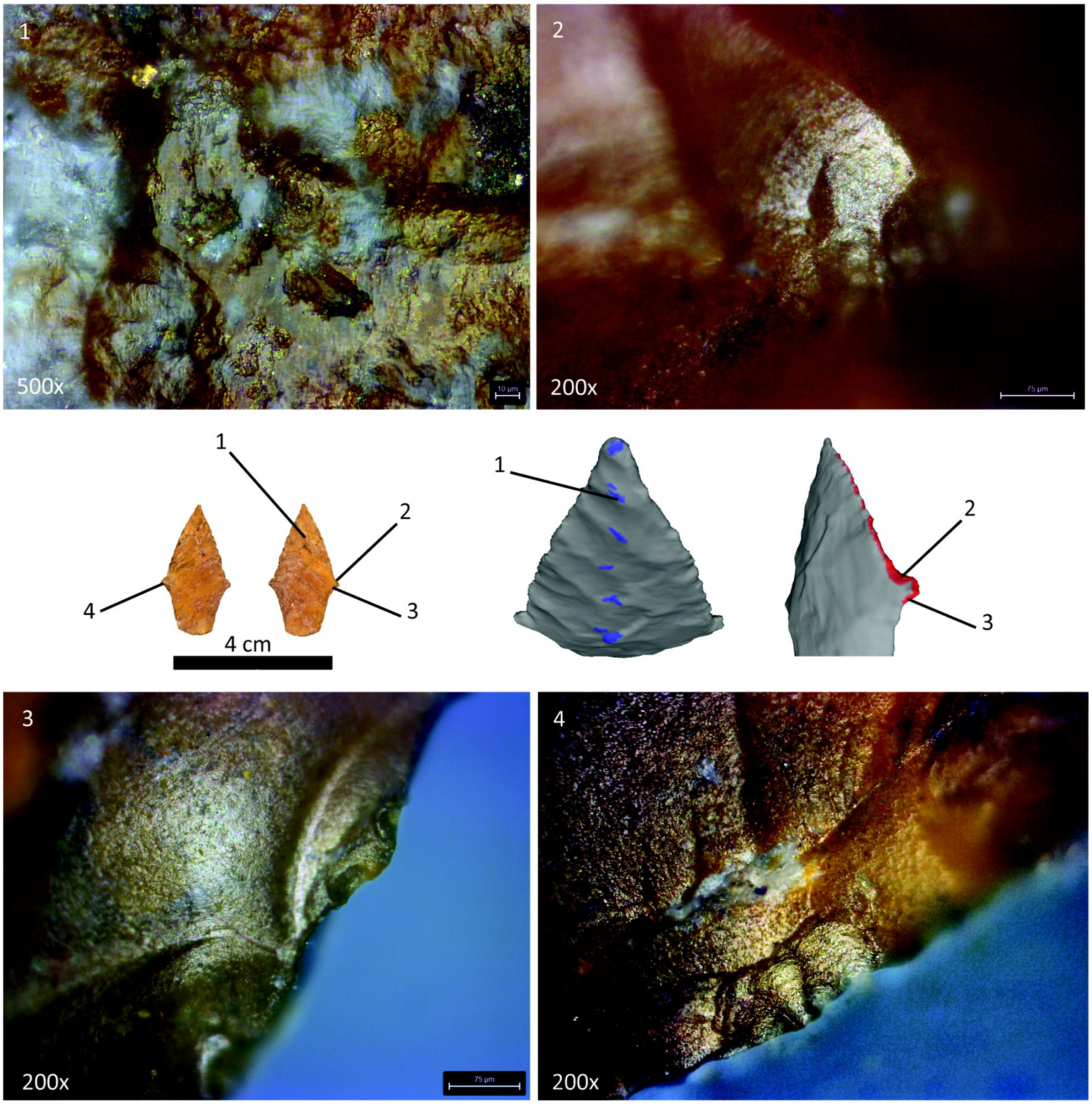

Fig. S4: WMP6-Artifact 2 acquired evidence of use as a projectile and knife. *1) Distal-facing facet of arris was polished from penetration and contact with hard material and faint parallel striae show the direction of motion. 2–4) Polish, rounding, and microflaking on distal and proximal-facing aspects of point ears from use as a knife.*

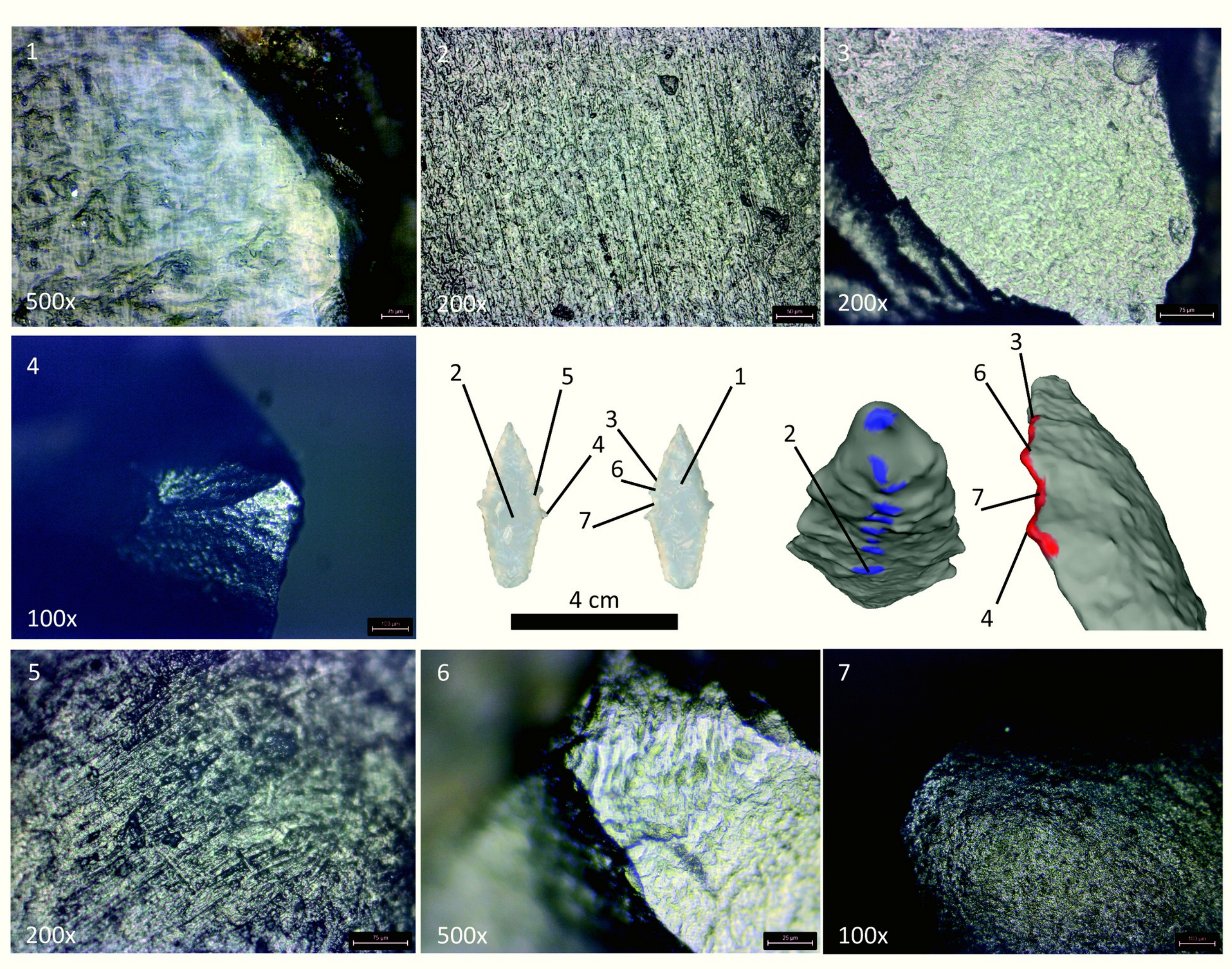

Fig. S5: WMP6-Artifact 3 acquired evidence of use as a projectile and knife. *1) Polish from use as a projectile was acquired on high facet on the microtopography along the point's center axis . 2) Striae located along and oriented parallel to the center axis show the direction of projectile motion. 3) Polish occurs along blade margin on a high flake scar arris from use as a knife. 4) An apex of point ear was polished from knife use. 5, 6) Striae on both faces of a point ear are oriented oblique to the blade margin, indicating this attribute was used in cutting. 7) A portion of the blade margin between protruding ears also acquired polish from use as a knife.*

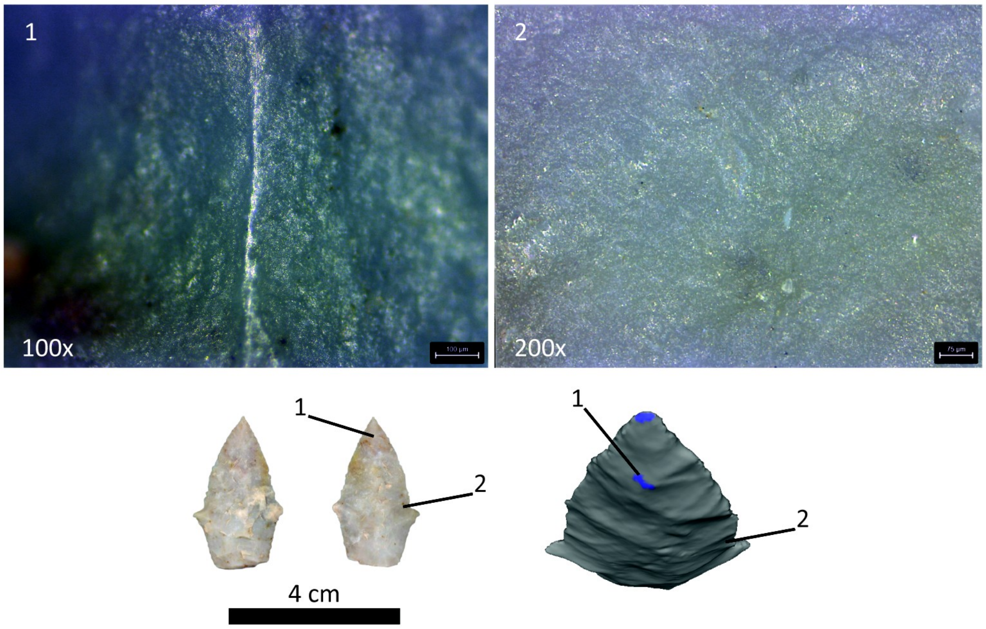

Fig. S6: WMP6-Artifact 4 acquired minimal evidence of use as a projectile. *1) A medial ridge along center axis was polished from impact. 2) No usewear traces along the blade margin indicate this point was not utilized as a knife.*

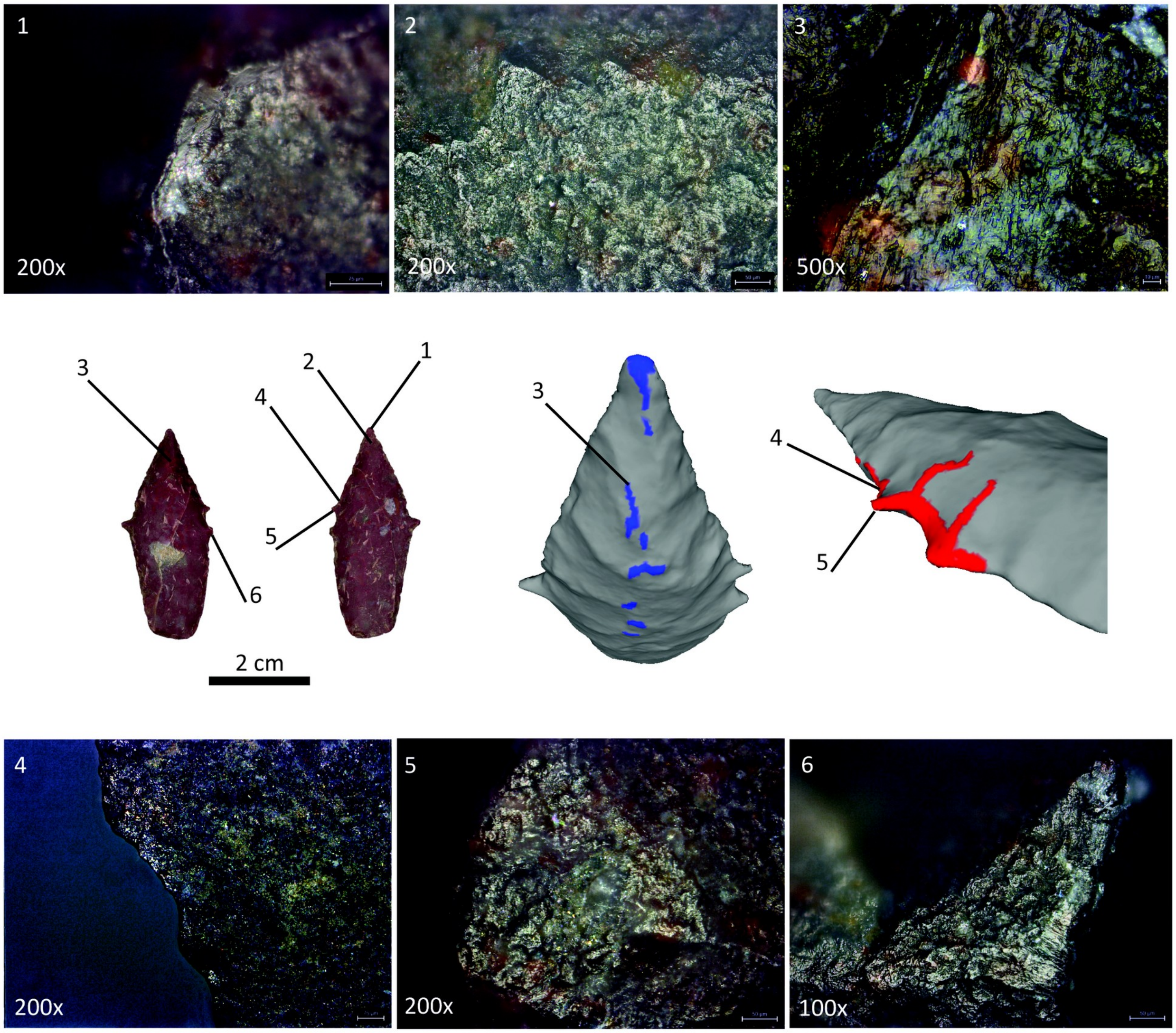

Fig. S7: WMP6-Artifact 5 acquired evidence of use as a projectile and knife. *1–3) Polish and parallel striae along the point's center axis indicate use as a projectile. 4) Polish along the blade edge, between small serrations, and down in negative flake scar are evidence of use as knife. 5, 6) Polish and a series of short striae oriented oblique to the lateral edge indicate the back of the ear contacted worked material in a cutting motion.*

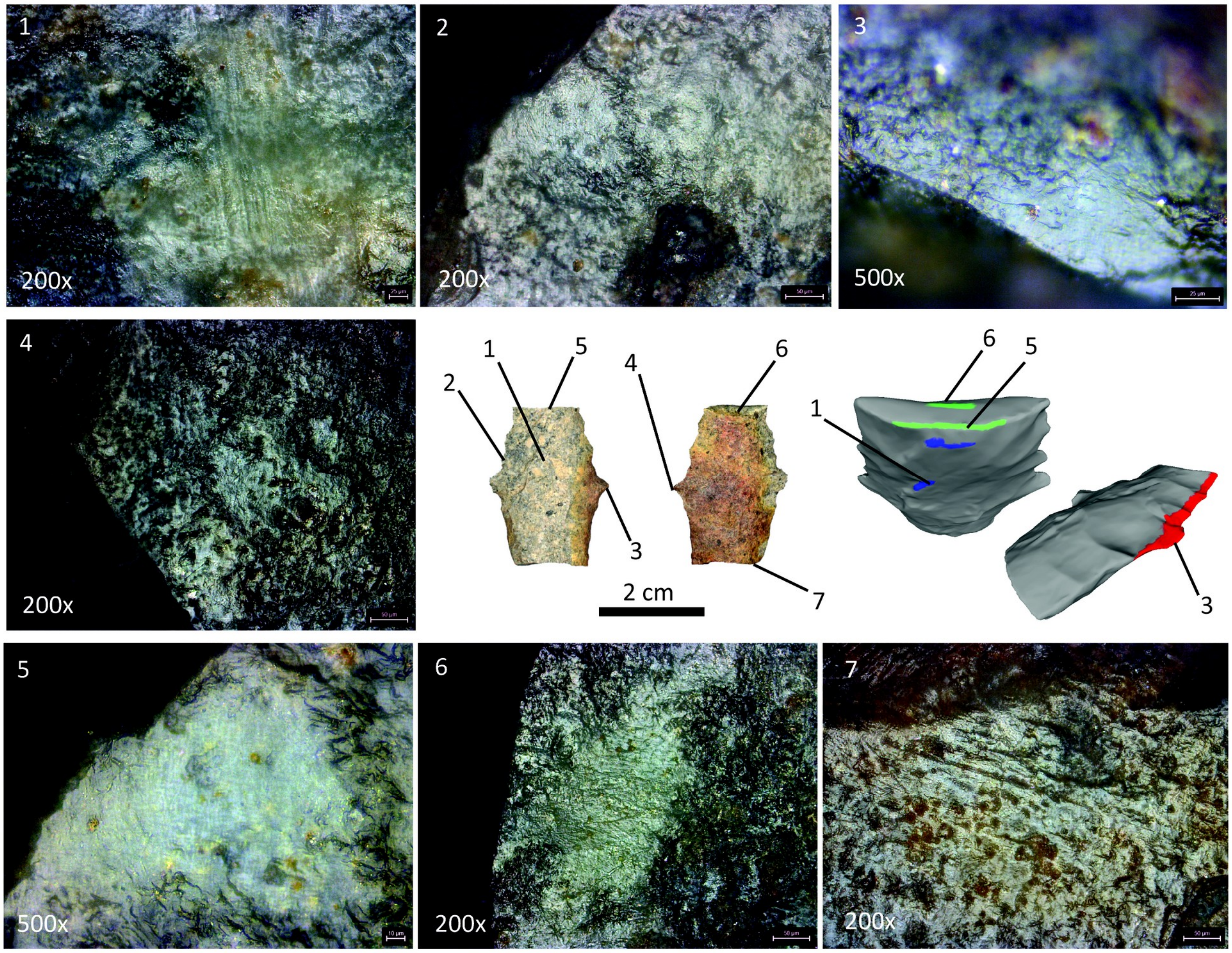

Fig. S8: WMP6-Artifact 6 acquired evidence of use as a projectile, knife, and scraper. *1) Striae and polish acquired along the center axis indicate the direction of motion with use as projectile. 2) Polish was acquired along the blade margin from use as knife. 3, 4) Apex and proximal surfaces of a point ear were polished from cutting. 5–7) Polish and perpendicular striae indicate the surfaces of hinge and snap breaks at the point's tip and base were used as scrapers.*

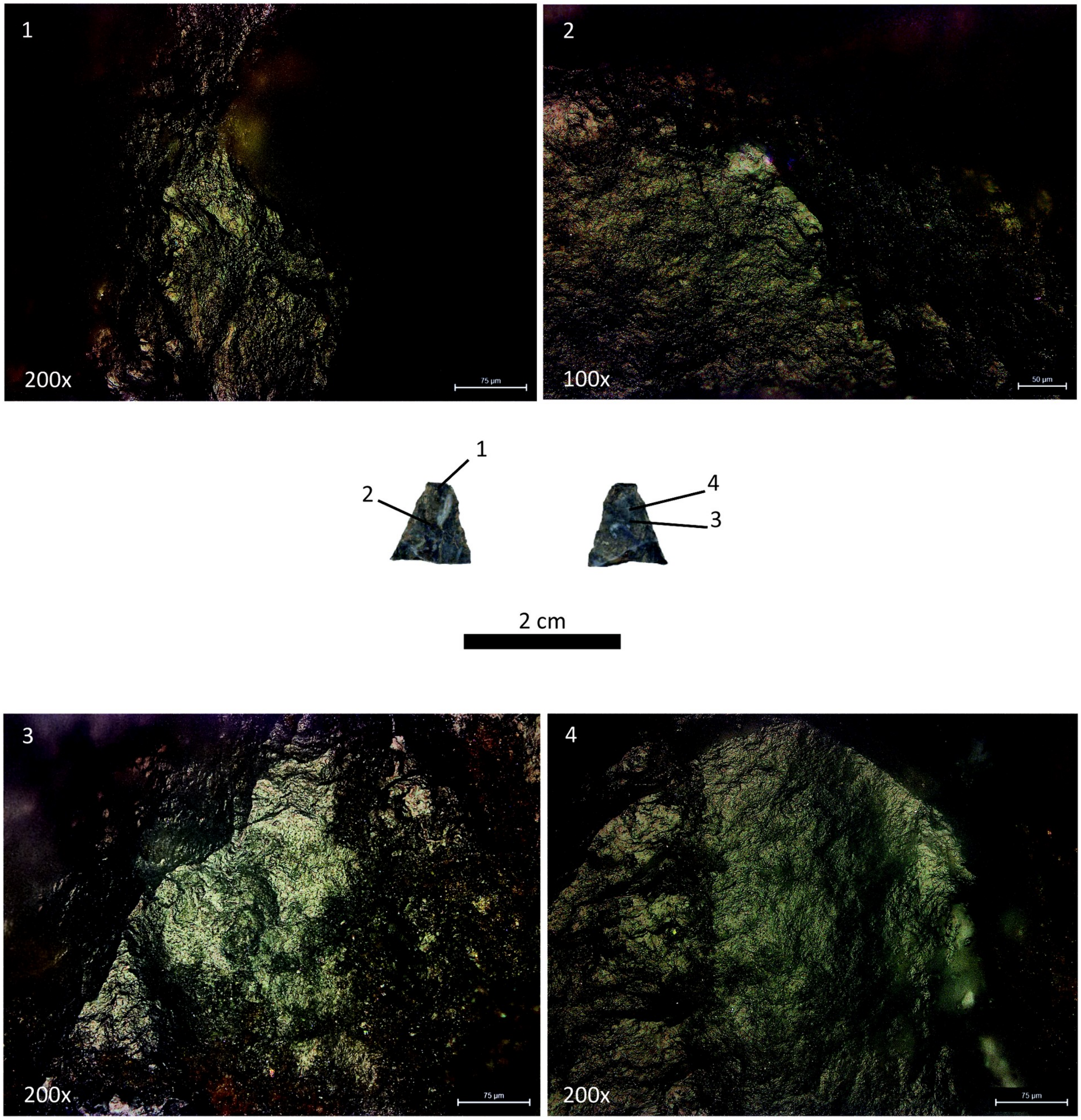

Fig. S9: WMP6-Artifact 7 acquired evidence of use as a projectile. *Polish occurs on high surfaces of the microtopography and distal-facing facets along the center axis.*

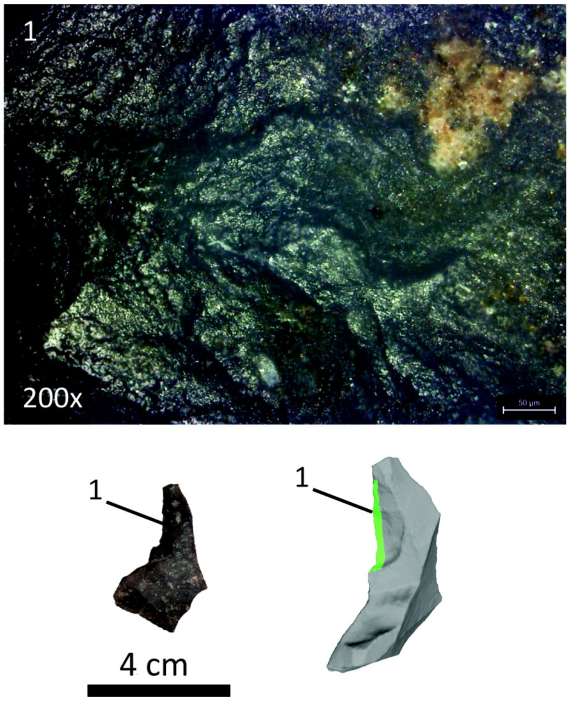

Fig. S10: WMP6-Artifact 8 acquired evidence of use from scraping. *Polish was acquired on low and high surfaces along the flake's concave edge.*

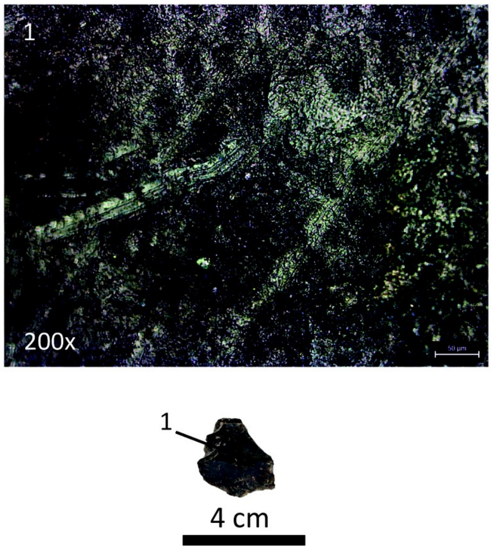

Fig. S11. WMP6-Artifact 9 acquired storage wear. *Unpatterned polish streaks caused by flake-on-flake damage.*

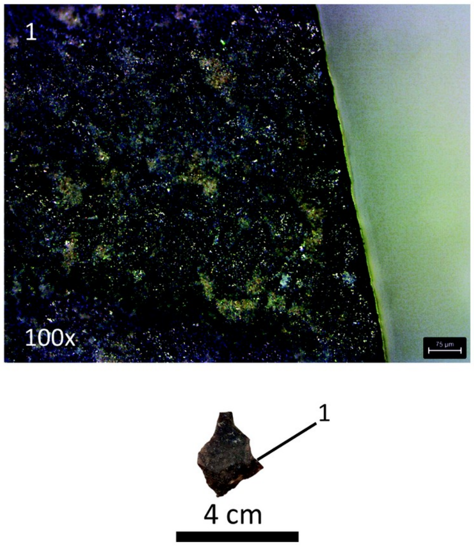

Fig. S12: WMP6-Artifact 10 shows no evidence of use. *Example of unused flake margin.*

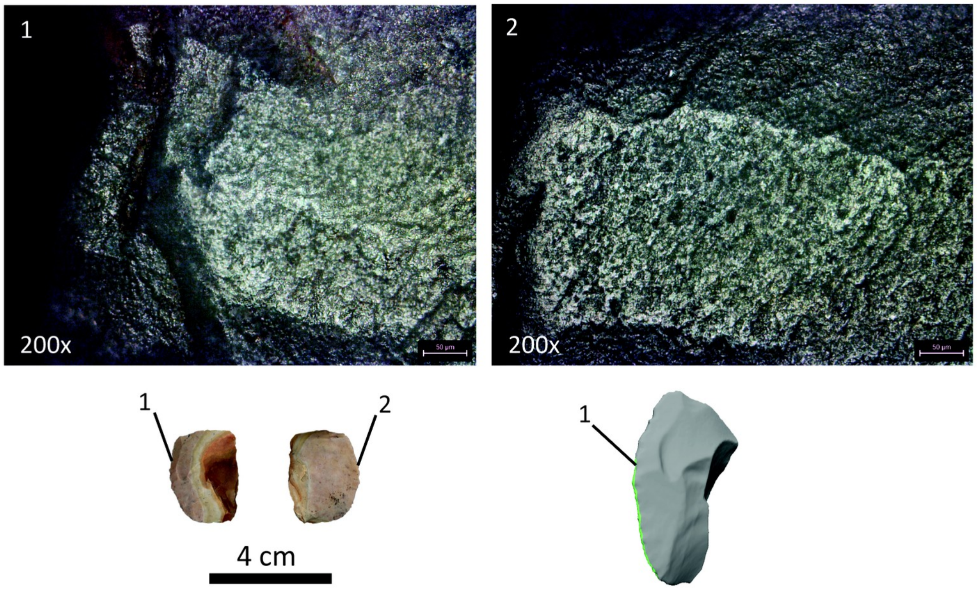

Fig. S13: WMP6-Artifact 11 acquired evidence of use from scraping. *High flake arrises along the margin acquired polish and stepped microflaking.*

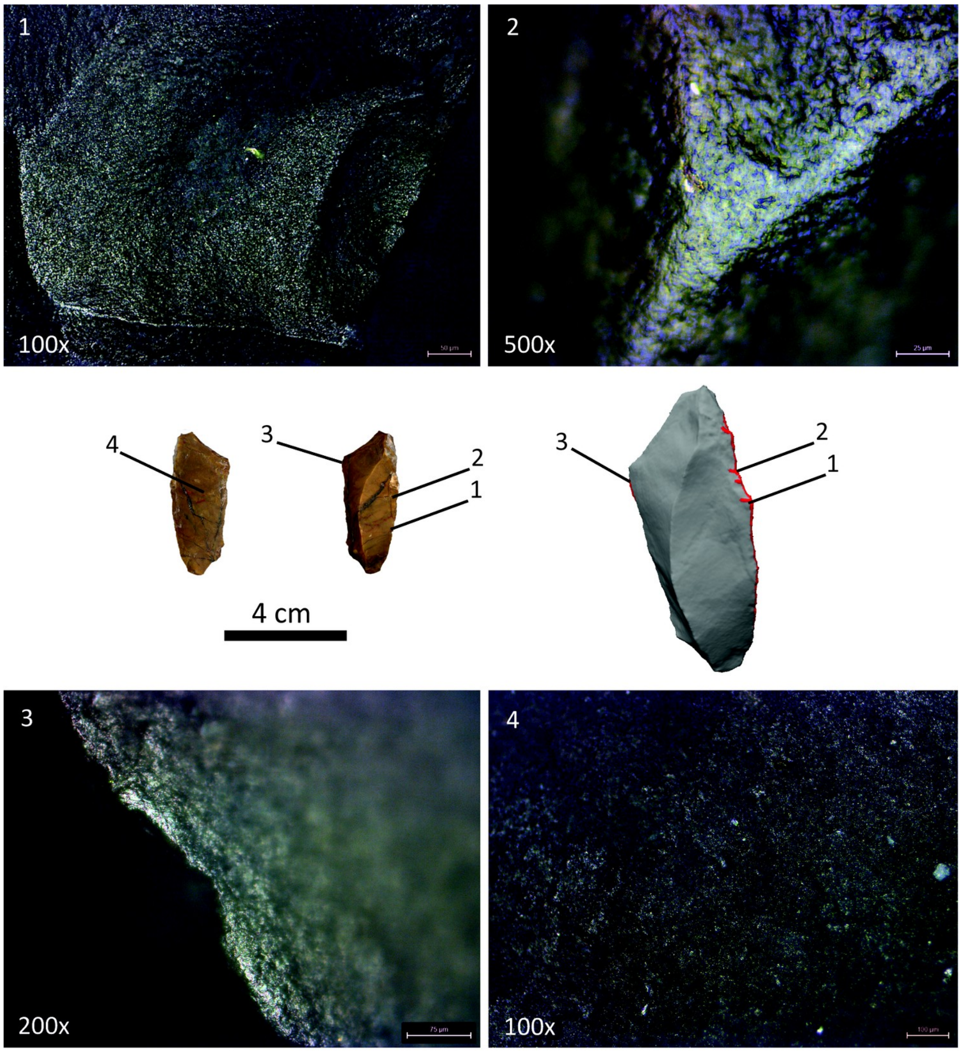

Fig. S14: WMP6-Artifact 12 acquired evidence of use from cutting. *1, 2) Polish acquired in negative flake scars and on high arrises of the modified flake margin indicate use in cutting. 3) A use unit on the opposite lateral margin also acquired polish. 4) The flake's interior ventral surface acquired no evidence of use, suggesting contact with worked material was restricted to flake margins.*

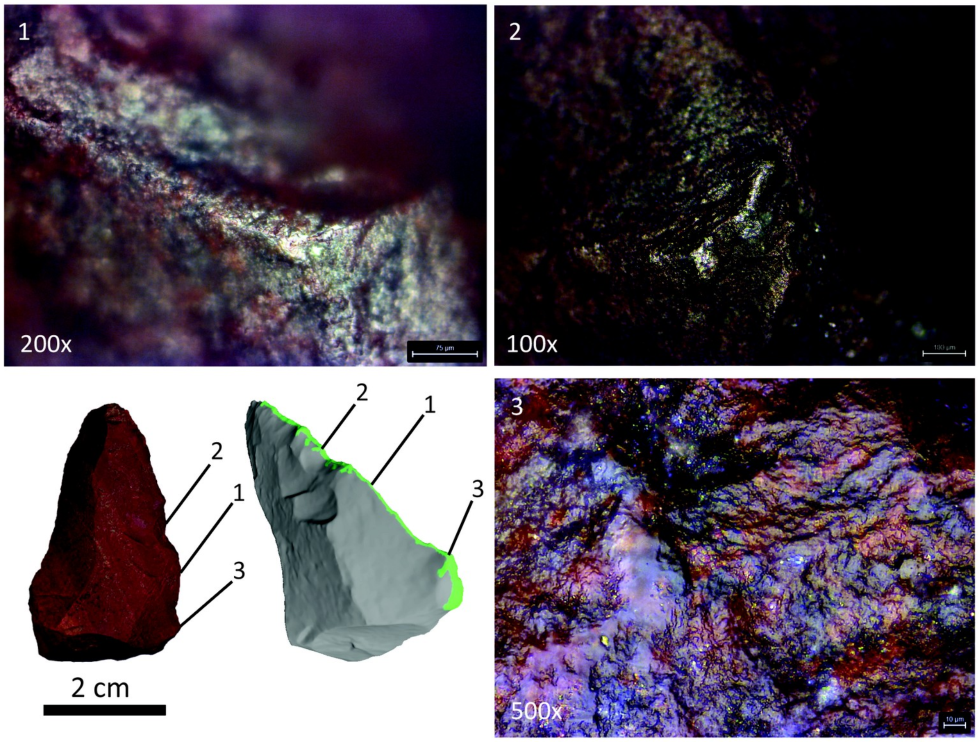

Fig. S15: WMP6-Artifact 13 acquired evidence of use from scraping. *Polish on flake scar arrises along the sinuous edge indicate this portion of the flake was used for scraping.*

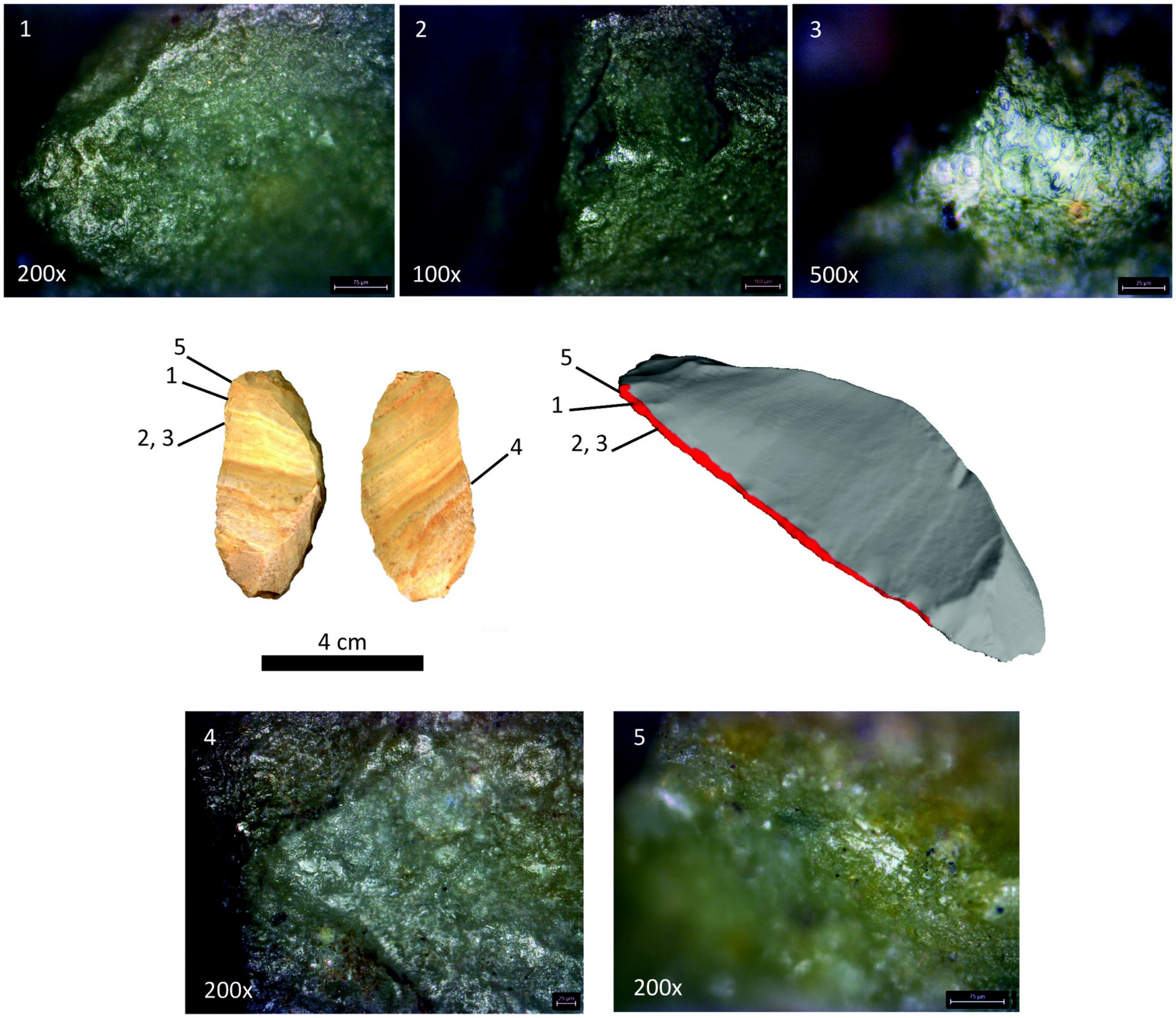

Fig. S16: WMP6-Artifact 14 acquired evidence of use as a backed knife. *1–4) Polish was acquired in low and high locations of the microtopography from use as a knife. 5) A striae oblique to the tool edge marks the proximal-most portion of the cutting margin.*

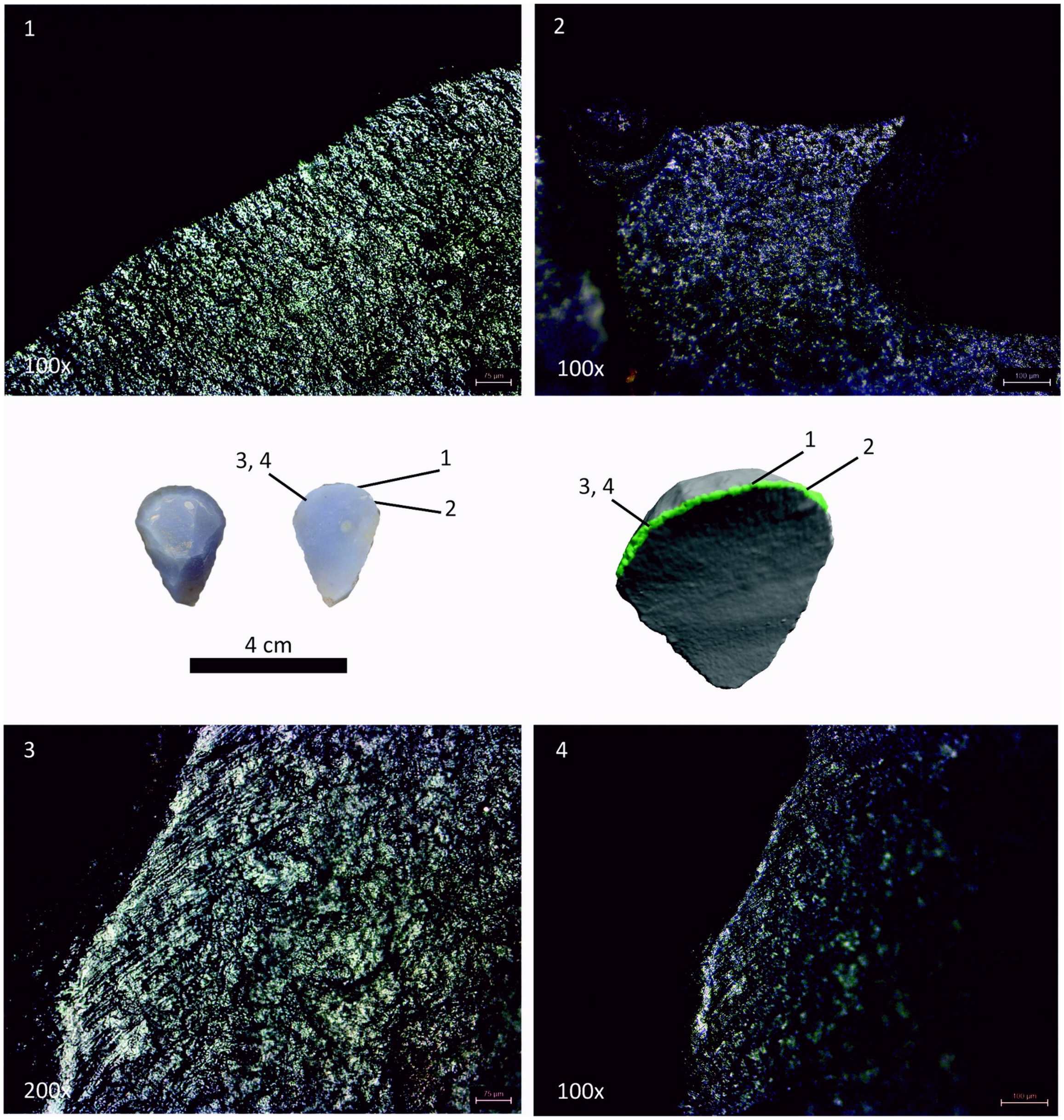

Fig. S17: WMP6-Artifact 15 acquired evidence of use as an endscraper. *1, 2) Edge rounding, polish, microchipping, and pitting along the ventral surface of the bit. 3, 4) Striae oriented oblique to the edge demonstrate the direction of scraping motion.*

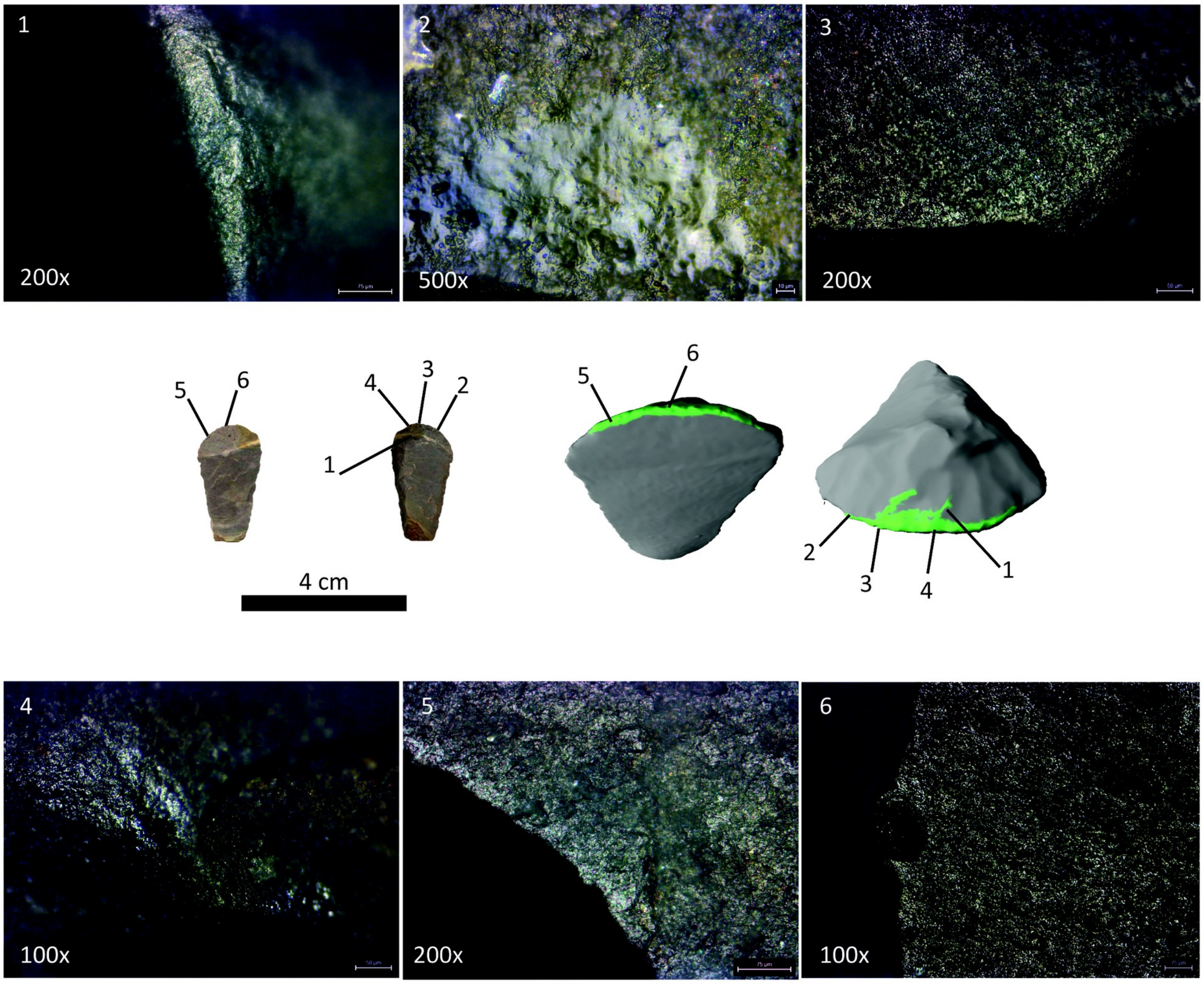

Fig. S18: WMP6-Artifact 16 acquired evidence of use as an endscraper. 1) Edge rounding, polish, microchipping, and pitting on flake scar terminations at the scraper bit indicate this surface contacted worked material. 2) Extensive polish with pitting was acquired in the center of the endscraper bit's dorsal surface. 3, 4) Polish and scalar microflaking occur on serial scalar flakes along the working edge. 5, 6) Polish and microflaking on the ventral surface are localized to the working edge.

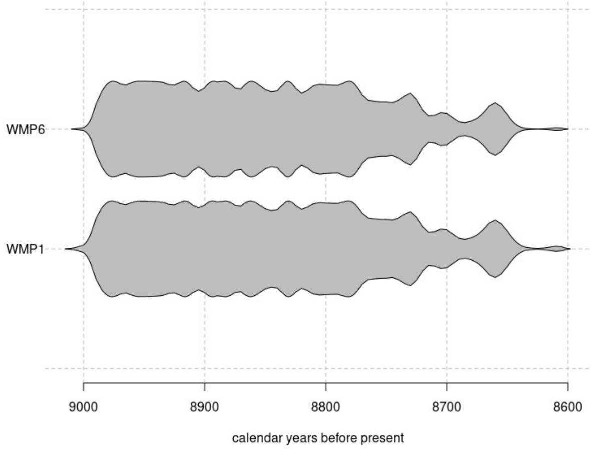

Fig. S19: Calibrated ^{14}C age estimates showing the virtual contemporaneity of the WMP 1 and WMP 6 individuals. *All dates are on bone collagen with good integrity. The WMP 1 date is based on a single sample producing an age estimate of 8010±25 ^{14}C BP.The WMP 6 date reflects an average of two statistically overlapping dates from the same individual, which produced an age estimate of 8008±16 ^{14}C BP using the date averaging method of Long and Rippeteau [17]. Both individuals produce calibrated 95% range estimates of 9.0–8.7 cal. BP. Calibration is based on the Shcal20 curve [18] as implemented using the Bchron package [19] in R statistical computing environment [20].*

References

1. Smallwood AM. Building experimental use-wear analogues for Clovis biface functions. Archaeological and Anthropological Sciences 2015;7:13-26. doi:10.1007/s12520-013-0139-2
2. Smallwood AM, Pevny CD, Jennings TA, Morrow JE. Projectile? Knife? Perforator? Using actualistic experiments to build models for identifying microscopic usewear traces on Dalton points from the Brand site, Arkansas, North America. Journal of Archaeological Science: Reports 2020;31:102337. doi:10.1016/j.jasrep.2020.102337
3. Haas R, Watson J, Buonasera T, Southon J, Chen JC, Noe S, Smith K, Llave CV, Eerkens J, Parker G. Female hunters of the early Americas. Science Advances 2020;6:eabd0310. doi:10.1126/sciadv.abd0310
4. Rots V. Wear traces and the interpretation of stone tools. Journal of Field Archaeology 2005;30:61-73. doi:10.1179/009346905791072404
5. van Gijn A. Functional differentiation of Late Neolithic settlements in the Dutch coastal area. In: . The interpretative possibilities of microwear studies: Proceedings of the international conference of use-wear analysis, 15th-17th February 1989 in Uppsala, Sweden. Upssala: Societas archaeologica Upsaliensis; 1990.
6. Chan B, Gibaja JF, Garca-Daz V, Hoggard CS, Mazzucco N, Rowland JT, van Gijn A. Towards an understanding of retouch flakes: A use-wear blind test on knapped stone microdebitage. PLOS ONE 2020;15:e0243101. doi:10.1371/journal.pone.0243101
7. Taipale N, Rots V. Every hunter needs a knife: Hafted butchering knives from Maisières-Canal and their effect on lithic assemblage characteristics. Journal of Archaeological Science: Reports 2021;36:102874. doi:10.1016/j.jasrep.2021.102874
8. Lurie R. A preliminary study of use-wear if the artifacts from some Preceramic components. In: MacNeish RS, Vierra RK, Nelken-Terner A, Garcia Cook A editors. Prehistory of the Ayacucho Basin, Peru: the Preceramic way of life (Vol. IV). Ann Arbor: University of Michigan Press; 1983.
9. Keeley LH. Experimental determination of stone tool uses: a microwear analysis. Chicago: University of Chicago Press; 1980.
10. Loebel TJ. Endscrapers, use-wear, and early Paleoindians in eastern North America. In: Gingerich JAM editor. In the Eastern fluted point tradition. Salt Lake City: University of Utah Press; 2013.
11. Miller GL, Bebber MR, Rutkoski A, Haythorn R, Boulanger MT, Buchanan B, Bush J, Lovejoy CO, Eren MI. Hunter-gatherer gatherings: stone-tool microwear from the Welling Site (33-Co-2), Ohio, U.S.A. supports Clovis use of outcrop-related base camps during the Pleistocene peopling of the Americas. World Archaeology 2018;51:47-75. doi:10.1080/00438243.2018.1461128
12. Waters MR, Pevny CD, Carlson DL, Dickens WA, Smallwood AM, Minchak SA, Bartelink E, Wiersema JM, Wiederhold JE, M.Luchsinger H, Alexander DA, Jennings TA. A Clovis workshop in central Texas: archaeological investigations of excavation area 8 at the Gault Site. College Station: Texas A&M Press; 2011.

13. Wiederhold JE, Pevny CD. Fundamentals in practice: a holistic approach to microwear analysis at the Debra L. Friedkin site, Texas. Journal of Archaeological Science 2014;48:104-119. doi:10.1016/j.jas.2013.07.035
14. Takase K. Use angle and motional direction of end scrapers: a case study of the Palaeolithic in Hokkaido, Japan. Asian Perspectives 2010;49:363-379. doi:10.1353/asi.2010.0013
15. Odell GH. The mechanics of use-breakage of stone tools: some testable hypotheses. Journal of Field Archaeology 1981;8:197. doi:10.2307/529414
16. Christidou R, Legrand-Pineau A. Hide working and bone tools: experimentation design and applications. In: Luik H, Choyke AM, Batey CE, Lõugas L editors. From hooves to horns, from mollusc to mammoth: manufacture and use of bone artefacts from prehistoric times to the present: proceedings of the 4th meeting of the ICAZ Worked Bone Research Group at Tallinn, 26th-31st of August 2003. Tallinn: Tallinn Book Printers Ltd; 2005.
17. Long A, Rippeteau B. Testing contemporaneity and averaging radiocarbon dates. American Antiquity 1974;39:205-215. doi:10.2307/279583
18. Hogg AG, Heaton TJ, Hua Q, Palmer JG, Turney CSM, Southon J, Bayliss A, Blackwell PG, Boswijk G, Ramsey CB, Pearson C, Petchey F, Reimer P, Reimer R, Wacker L. SHCal20 southern hemisphere calibration, 0–55,000 years cal BP. Radiocarbon 2020;62:759-778. doi:10.1017/rdc.2020.59
19. Haslett J, Parnell AC. A simple monotone process with application to radiocarbon-dated depth chronologies. Journal of the Royal Statistical Society: Series C (Applied Statistics) 2008;57:399-418. doi:10.1111/j.1467-9876.2008.00623.x
20. R Core Team. R: A Language and Environment for Statistical Computing. Vienna. Vienna, Austria 2022.
